# Machine Learning Prediction of Porosity in Low-Pressure-Cast AlSi7Mg Alloy with Varying Recycled Aluminum Content

**DOI:** 10.3390/ma19214729

**Published:** 2026-11-06

**Authors:** Jaroslaw Piatkowski, Grzegorz Siwiec, Tomasz Matula, Beata Oleksiak, Karel Gryc, Andrzej Grabowski, Katarzyna Nowinska

**Affiliations:** 1 Materials Innovation Laboratory, Faculty of Materials Science and Industrial Digitalization, Silesian University of Technology, Krasinskiego 8, 40-019 Katowice, Poland; jaroslaw.piatkowski@polsl.pl; 2 Department of Metallurgy and Recycling, Faculty of Materials Engineering and Industrial Digitalization, Silesian University of Technology, Krasińskiego 8, 40-019 Katowice, Poland; tomasz.matula@polsl.pl; 3 Department of Production Engineering, Faculty of Materials Science, Silesian University of Technology, Krasinskiego 8, 40-019 Katowice, Poland; beata.oleksiak@polsl.pl; 4 Environmental Research Department, Institute of Technology and Business in České Budějovice, Okružní 517/10, 370 01 České Budějovice, Czech Republic; gryc@mail.vstecb.cz; 5 Institute of Physics—Centre for Science and Education Silesian, University of Technology, Krasińskiego 8, 40-019 Katowice, Poland; andrzej.grabowski@polsl.pl; 6 Faculty of Mining, Safety Engineering and Industrial Automation, Silesian University of Technology, Ul. Akademicka 2, 44-100 Gliwice, Poland; katarzyna.nowinska@polsl.pl

**Keywords:** AlSi7Mg, recycled aluminum, low-pressure casting, porosity, Fe–Mn, machine learning, explainable AI, sludge factor

## Abstract

Industrial AlSi7Mg produced by low-pressure casting was evaluated with 50–95 wt.% recycled material under two conditions: no intentional Mn correction and Mn correction targeted at Mn/Fe ≈ 0.5. The porosity dataset contained 200 observations; for 60–95 wt.% recycled material, 160 records from 16 experimental conditions also included sample-specific chemical composition. Without Mn correction, MeanPor rose from 0.160% to 6.111%, whereas Mn correction reduced porosity under most conditions. Eight hypothesis-driven candidate feature architectures and 13 model families were assessed by grouped Leave-One-Scrap-Level-Out cross-validation, with hyperparameters tuned only on training data. A second-order regression with predefined interactions using recycled material content, Mn-correction variant, and sample-specific Fe gave the lowest out-of-fold (OOF) error for MeanPor (coefficient of determination (R^2^) = 0.9910, root mean square error (RMSE) = 0.1691, mean absolute error (MAE) = 0.1508). The reduced Sludge Factor descriptor, *SF*_*Cr*0_ = (1 × %Fe) + (2 × %Mn), also improved prediction and reached R^2^ ≈ 0.986. SHAP (SHapley Additive exPlanations) was used to describe model attribution without causal interpretation. At 90 and 95 wt.% recycled material, Mn correction coincided with higher V values and more regular pore cross-sections. Sample-specific chemistry improved porosity prediction within the investigated process window; independent validation is required before use outside that range.

## 1. Introduction

Aluminum alloys are widely used in transportation and engineering applications because of their low density, favorable strength-to-weight ratio, castability, and recyclability [1,2,3]. Increasing the proportion of recycled aluminum in foundry charge provides substantial environmental and energy benefits [4,5]. However, a higher recycled-material content also makes control of melt chemistry and liquid-metal quality more demanding because residual and tramp elements can accumulate during repeated recycling cycles. In Al-Si casting alloys, Fe is particularly important because of its low solubility in α(Al) and its tendency to form Fe-rich intermetallic compounds with morphologies that may adversely affect casting quality [2,3,6,7,8].

Porosity is one of the major quality concerns in aluminum castings. Its formation cannot generally be attributed to a single mechanism. Solidification shrinkage, insufficient feeding, dissolved hydrogen, oxide films, and other inclusions can all contribute to pore formation [9,10,11,12,13,14,15,16]. Fe-rich intermetallic phases may additionally influence local feeding and defect development, while the interaction between intermetallic phases and porosity can affect mechanical properties [14,17]. Consequently, increasing recycled-material content may alter porosity both directly through changes in melt quality and indirectly through changes in chemical composition and solidification behavior. In the present industrial system, increasing recycled aluminum content was accompanied by a systematic increase in Fe content.

Mn addition is frequently used to modify the morphology of Fe-rich intermetallic phases in Al-Si alloys. It can reduce the prevalence of plate-like β-Al5FeSi-type morphologies and promote more compact α-Al(Fe,Mn)Si-type morphologies, although the resulting microstructure depends on Fe and Mn contents and solidification conditions [7,18,19,20,21]. Fe, Mn, and Cr are also commonly combined in the Sludge Factor as an empirical descriptor of the tendency to form heavy Fe-rich compounds [22,23]. However, threshold values established for other casting technologies cannot be transferred directly to low-pressure casting. This makes it relevant to compare the predictive information contained in measured Fe itself with that contained in a combined Fe–Mn descriptor.

The practical challenge is therefore not only to describe how porosity changes with recycled-material content, but also to determine whether porosity can be predicted from information available before or during casting. A simple relationship with recycled-material content alone may be insufficient because recycled content, Fe level, Mn correction, and other chemical variables change simultaneously. Machine-learning and regression methods provide a framework for evaluating such interacting predictors, but small material datasets are particularly vulnerable to overfitting and optimistic validation [24,25]. Consequently, the validation strategy is as important as the model algorithm itself. Recent studies have demonstrated the use of data-driven methods for porosity, microstructure, mechanical-property design, and Fe-rich-phase prediction in aluminum alloys [26,27,28,29,30], while explainable-AI methods can help describe the predictive information used by fitted models [31]. However, model explanations should not be interpreted automatically as causal metallurgical mechanisms.

The specific question addressed in this study is whether porosity in industrial low-pressure-cast AlSi7Mg with a high recycled-aluminum content can be predicted from the recycled-material level, Mn-correction state, and sample-specific melt chemistry, and whether measured chemical composition provides additional predictive information beyond process variables alone. The full experimental campaign comprised recycled-material contents from 50 to 95 wt.%. Chemistry-enhanced modeling was restricted to 60–95 wt.%, for which sample-specific chemical composition was available.

Eight hypothesis-driven candidate feature architectures and thirteen model families were compared using outer-grouped Leave-One-Scrap-Level-Out cross-validation. Mean pore area fraction (MeanPor) was the primary response, while the most porous field (MostPor) and mean pore cross-sectional area (PoreArea) were analyzed as supplementary responses. Measured Fe was compared with the reduced descriptor *SF*_*Cr*0_ = (1 × %Fe) + (2 × %Mn). The study therefore combines industrial casting experiments, quantitative metallography, pore-morphology analysis, chemical characterization, machine-learning prediction, and model explanation while explicitly separating predictive performance from mechanistic interpretation.

## 2. Materials and Methods

### 2.1. Experimental Design and Material System

The study was conducted on an industrial AlSi7Mg casting alloy intended for low-pressure casting (LPC). The experimental plan was designed to assess the effects of increasing recycled material content and Mn correction on chemical composition, porosity, and pore morphology, and subsequently to build and interpret predictive models using regression methods, machine learning, and explainable artificial intelligence (XAI). The recycled material content in the charge was varied from 50 to 95 wt.% in increments of 5 percentage points. Two alloy variants were prepared at each level: without intentional Mn correction and after Mn correction targeted at Mn/Fe ≈ 0.5. The term “without Mn correction” denotes the absence of an intentional Mn addition rather than the complete absence of Mn; small residual amounts were present in the alloy. For each of the 20 conditions, 10 separate melts were run and one wheel casting was sampled from each (*n* = 10), yielding 200 porosity observations. The extended chemistry–process modeling was restricted to the range of 60–95 wt.% recycled material because, within this common range, sample-specific chemical-composition results were available for the same trials in which porosity was measured. The chemical composition + porosity dataset therefore comprised 160 complete observations.

The recycled fraction consisted exclusively of internal return scrap generated within the company and returned to production. The main streams comprised castings from pre-production/test batches, castings rejected during final quality control because of internal or external defects or failure to meet required mechanical properties, machining chips, and gating-system elements such as risers, overflows, and runners. No externally purchased scrap was used in the investigated campaign. Recycled material content was defined as the mass percentage of the total charge, with the balance supplied by primary material; for the nominal 20 t melt mass, this corresponded to 10–19 t of recycled material over the investigated 50–95 wt.% range.

10 scrap levels × 2 Mn variants × 10 trials = 200 porosity observations

8 scrap levels × 2 Mn variants × 10 trials = 160 paired chemistry + porosity records

Each record represented one independent experimental trial corresponding to one casting. A sample was taken from the tread area of each wheel casting; one casting corresponded to one observation in the dataset. Ten randomly selected fields of view were subsequently analyzed for each sample; these fields were subsamples used to calculate the porosity descriptors for that casting and were not treated as separate model records. Chemical-composition and porosity results were linked using the corresponding Measurement_i identifiers. Individual results were not replaced by condition means, missing compositions were not imputed, and synthetic replicates were not generated. The 160 chemistry–porosity records therefore represented 160 independently produced castings nested within 16 experimental conditions (8 recycled-material levels × 2 Mn variants). At the process-factor level, however, the chemistry-resolved analysis contained only eight distinct recycled-material levels. Sample-specific chemistry was not available at 50 and 55 wt.% recycled material; these two levels were used only in the full-range descriptive porosity analysis and were not included in chemistry-enhanced model fitting. The experimental design and datasets are summarized in Table 1.

### 2.2. Chemical Composition and Sludge Factor

Industrial AlSi7Mg alloy was used as the base material. The nominal chemical-composition range of the base alloy is given in Table 2.

Increasing recycled material content was accompanied by changes in alloy chemistry, particularly an increase in Fe content. In the series without intentional Mn correction, mean Fe increased from approximately 0.13 wt.% at 60 wt.% scrap to approximately 0.69 wt.% at 95 wt.% scrap. In the Mn-corrected series, the Mn/Fe ratio was maintained close to 0.5; for example, mean Mn was approximately 0.068 wt.% at 60 wt.% scrap and approximately 0.355 wt.% at 95 wt.% scrap. Chemical analysis included Si, Fe, Cu, Mn, Mg, Zn, Ti, Ca, Sr, and the sum of the remaining measured constituents. Chemical-composition control was performed using a SpectroLab emission spectrometer (SPECTRO Analytical Instruments GmbH, Kleve, Germany) on liquid-metal samples taken during melting/holding and before casting. The density index was additionally monitored by the hydrostatic method, with 1–1.5% treated as the acceptable process-control range.

The Sludge Factor was used as a separate chemical descriptor. Its full form for secondary Al-Si alloys is defined as follows [32]:

*SF* = (1 × %Fe) + (2 × %Mn) + (3 × %Cr) (1)

Cr was not intentionally added to the investigated alloys, and its sample-specific residual content was not measured. Therefore, the reduced descriptor *SF*_*Cr*0_ = (1 × %Fe) + (2 × %Mn) was defined.

*SF*_*Cr*0_ = (1 × %Fe) + (2 × %Mn) (2)

The *Cr0* subscript was retained to avoid implying an analytically confirmed absence of Cr. The “other” category was not treated as a substitute for Cr content. For each trial, *SF*_*Cr*0_ was determined individually from the measured Fe and Mn contents. *SF*_*Cr*0_ was treated solely as a reduced Fe–Mn descriptor rather than the full Sludge Factor; its absolute values were not compared directly with the literature thresholds established for the full *SF* = (1 × %Fe) + (2 × %Mn) + (3 × %Cr). Because *SF*_*Cr*0_ is deterministically constructed from Fe and Mn, it was evaluated in separate input architectures and was not interpreted as an independent causal mechanism.

### 2.3. Liquid-Metal Preparation and Low-Pressure Casting

After the proportions of primary and recycled material had been prepared, the alloy was melted under industrial conditions. The melt temperature in the melting furnace was approximately 740 °C. The alloy was then transferred to a ladle preheated to approximately 200 °C, where AlTi5B1 grain refinement (Ti/B = 5:1; target Ti addition approximately 0.05 wt.%) and nitrogen refining were performed. Nitrogen was introduced by bubbling at 18.5–19.5 L/min for approximately 10 min. After ladle treatment, the melt was transferred to an electric holding furnace and maintained continuously at approximately 690–700 °C. The LPC cycle used three successive pressure stages of approximately 200, 500, and 800 mbar (20, 50, and 80 kPa). The casting was cooled with water and the die with compressed cold air; the typical casting-cooling time was 10–20 s depending on casting size. The casting temperature after removal from the die was approximately 400 °C, after which the casting was water-cooled to approximately 30–40 °C. Finished castings subsequently underwent X-ray inspection, T6 heat treatment (solution treatment and artificial aging), and further surface-finishing operations. These process parameters, summarized in Table 3, were maintained according to the fixed industrial production procedure and were not experimental predictors in the present dataset. Separate numerical values for filling time and pressure-holding duration were not retained in the research documentation and are therefore not introduced retrospectively.

For the Mn-corrected series, AlMn20 master alloy containing 20 wt.% Mn was used in rod form. It was added during melting in an amount selected to obtain a target Mn/Fe ratio of approximately 0.5 and was introduced approximately 15 min before casting; this interval had been established in preliminary trials to allow dissolution and homogenization before casting. The resulting composition was verified spectrometrically. In addition, crystallization curves were recorded for selected samples by thermal-derivative analysis (ATD), monitoring characteristic temperatures including Tliq, TE, TEMn, and Tsol under the same measurement conditions. ATD served as an auxiliary process check and was not used as a predictor in the machine-learning dataset.

### 2.4. Metallographic Preparation and Microscopy Analysis

Metallographic specimens were prepared according to the recommendations of the Buehler expert system. Sectioning was performed using a Delta AbrasiMet Cutter, model 102511 (Buehler, Lake Bluff, IL, USA). The principal quantitative porosity measurements and light-microscopy (LM) observations were carried out using an Olympus GX71MeF2 microscope (Olympus Global EMC, Ltd., Tipton, UK). For each of the 10 independent castings under a given condition, a sample was taken from the tread area, and 10 images were recorded for each sample in randomly selected fields of view. Quantitative porosity analysis was performed on unetched specimens (Table 4). Some specimens were etched with Fuss reagent solely for qualitative microstructural observations. Selected representative specimens at 70, 80, and 90 wt.% recycled material were additionally examined by scanning electron microscopy (SEM) using a Hitachi S-3400N (Hitachi High-Tech Corporation, Tokyo, Japan) in backscattered-electron mode at 200×. The observed constituents were assessed morphologically; no EDS or diffraction-based identification of individual phases was performed. Consequently, phase assignments in the subsequent interpretation refer to characteristic morphologies reported in the literature rather than to direct confirmation of phase stoichiometry.

The specimens used for quantitative porosity measurements were flat coupons sectioned from the tread region of each wheel. Measurements were performed on the polished, unetched flat cross-sectional surface. The archived preparation records did not retain the overall coupon dimensions or an additional radial/circumferential orientation label relative to the wheel axis; therefore, no unverified dimensions or orientation were introduced retrospectively.

### 2.5. Quantitative Porosity Analysis and Shape Factor V

Quantitative porosity analysis was performed in AnalySis 12.2 (Olympus Soft Imaging Solutions GmbH, Münster, Germany) using quantitative metallography and bright-field image analysis on unetched specimens. Ten randomly selected fields of view were analyzed for each sample. Pores were segmented using the Otsu method, and area-based measurements were performed on 2D sections. MeanPor denoted the mean pore area fraction calculated from the 10 analyzed fields. MostPor was defined as the highest porosity area fraction among these 10 fields. PoreArea denoted the mean cross-sectional area of all pores identified in the 10 fields of a given sample. MeanPor was the primary target variable, whereas MostPor and PoreArea were analyzed as auxiliary variables. The response variables and the morphology descriptor V are summarized in Table 5.

Quantitative analysis was performed at 200× magnification with an image calibration of approximately 1.0309 µm/pixel. The quantitative region of interest was 606 × 400 pixels, excluding the lower scale-bar strip, corresponding to approximately 0.258 mm^2^ per field and 2.576 mm^2^ for the ten fields analyzed per specimen. The minimum accepted object size was 1 pixel (approximately 1.1 µm^2^). The Otsu threshold was determined automatically and independently for each image from its grayscale intensity distribution, without manual adjustment. For MeanPor and the field values used to determine MostPor, the visible fraction of pores intersecting an image boundary was retained. For individual-pore PoreArea and V measurements, boundary-touching objects were excluded because their complete area and perimeter were not available. The ten randomly selected fields constituted the fixed sampling protocol used consistently for all specimens; MostPor should therefore be interpreted as the maximum among these ten sampled fields, not as the absolute maximum porosity anywhere in the wheel.

Pore morphology was additionally described by the dimensionless shape factor V, corresponding to the coefficient ξ defined by Roskosz [33]: V = 4πA/P^2^, where A is the pore cross-sectional area and P is its perimeter. Values closer to 1 correspond to more regular and rounded cross-sections. In the documentation used for the investigated LPC AlSi7Mg system, V = 0.8 was adopted as an auxiliary morphological criterion: values of V = 0.8–1.0 were generally associated with more regular cross-sections, whereas V < 0.8 was associated with more irregular ones. The value 0.8 was treated solely as an auxiliary, condition-dependent morphological criterion rather than a universal boundary for classifying the pore-formation mechanism.

For each complete pore object, V was calculated from its measured area and perimeter. Specimen-level V was calculated as the unweighted arithmetic mean of pore-by-pore V values pooled across the ten analyzed fields. Condition-level Mean V and SD(V) were then calculated from the ten independent casting-level V observations for each condition; no area weighting was applied between castings. A representative original 200× LM image and the corresponding Otsu binary mask illustrate the segmentation workflow.

### 2.6. Modeling Dataset and Predictor Architectures

Chemistry–process modeling was performed on 160 paired records. The variable Scrap (S) was defined as recycled material content [wt.%], whereas the Mn-correction variant was encoded as M = 0 for the series without intentional Mn correction and M = 1 for the Mn-corrected series. MeanPor was the primary target; MostPor and PoreArea were analyzed as auxiliary targets. To separate process information from chemical information, eight hypothesis-driven candidate input architectures were specified on the basis of metallurgical rationale rather than automated feature selection. The study was not preregistered; accordingly, these architectures should be regarded as hypothesis-driven candidate representations rather than preregistered specifications. They were defined before the final comparison of the 13 model families. The predictor architectures and their roles are summarized in Table 6.

Selected quadratic terms and interactions were used in the explicit models. Model B included S, M, Fe, S^2^, S × M, and Fe × M, whereas model E included S, M, *SF*_*Cr*0_, S^2^, S × M, and *SF*_*Cr*0_ × M. Higher-order interactions were not introduced automatically in order to limit the number of model parameters and the risk of overfitting, given that the chemical dataset contained only eight recycled-material levels.

### 2.7. Algorithms, Preprocessing, and Hyperparameter Tuning

Thirteen model families were compared within each architecture: second-order regression with predefined interactions, Polynomial Ridge, partial least squares (PLS), Gaussian process regression with a radial basis function kernel (GPR-RBF), GPR with a Matérn 5/2 kernel (GPR-Matérn 5/2), Kernel Ridge with an RBF kernel, and support vector regression with an RBF kernel (SVR-RBF), Random Forest, Extra Trees, Gradient Boosting, XGBoost, CatBoost, and multilayer perceptron (MLP). For methods sensitive to input scale, standardization was performed exclusively on the training data of a given fold, and the transformation parameters were then applied without refitting to the validation or test data. For models evaluated using discrete hyperparameter sets, configuration selection was carried out exclusively within the inner grouped validation. The tuning ranges were limited a priori because of the sample size and grouped structure of the dataset (Table 7).

For Gaussian process regression, automatic relevance determination RBF (ARD-RBF) and ARD-Matérn 5/2 kernels with a WhiteKernel term were used. Kernel parameters were optimized by maximizing the log marginal likelihood without additional optimizer restarts. This setting was treated as a deliberately constrained benchmark variant chosen to limit procedural degrees of freedom given the small number of independent process levels.

### 2.8. Nested Grouped Validation

Because of the grouped structure of the data, neither a random train/test split nor random k-fold validation was used. Such a split could place observations belonging to the same nominal recycled-material level in both the training and test sets, resulting in an overly optimistic assessment of generalization. Outer-grouped cross-validation was performed using Leave-One-Scrap-Level-Out (LOSO). In each of the eight iterations, one entire recycled-material level—comprising 10 observations without Mn correction and 10 observations after Mn correction—was excluded from training and used as the outer test set. The outer training set contained 140 observations from seven levels, whereas the outer test set contained 20 observations from one unseen level. Within each outer training set, models requiring tuning were optimized using 3-fold GroupKFold, again grouping by recycled-material level. Hyperparameters were selected exclusively on the basis of inner CV; the model was then fitted to the complete outer training set and used to generate predictions for the unseen outer test set. Across the eight iterations, each of the 160 observations received exactly one out-of-fold (OOF) prediction, and the primary performance metrics were calculated from the combined set of OOF predictions. This procedure evaluates generalization to an unseen recycled-material level within the present experimental campaign. It does not constitute independent external validation across different production campaigns, equipment, time periods, laboratories, or manufacturing sites.

8 outer folds × 20 test observations = 160 OOF predictions

The feature architectures were hypothesis-driven candidate representations based on the research questions and metallurgical rationale, and no automated response-based feature-selection procedure was used. At the same time, the same outer-CV results were used to compare multiple algorithm families. Therefore, the metrics of the model identified as best should be interpreted as within-study outer-grouped cross-validation of individual candidates within the defined modeling experiment, rather than as a fully independent evaluation of the entire winner-selection procedure or as external industrial validation.

### 2.9. Prediction Metrics, Statistics, and Bootstrap

Root mean square error (RMSE) was the primary ranking metric; the coefficient of determination (R^2^) and mean absolute error (MAE) were additionally reported.

(3) $$\mathrm{RMSE}=\surd [(1/n)\;\Sigma {\left(y_{i}-{\hat{y}}_{i}\right)}^{2}]$$

(4) $$\mathrm{MAE}=(1/n)\;\Sigma |y_{i}-{\hat{y}}_{i}|$$

(5) $$R^{2}=1-\Sigma {\left(y_{i}-{\hat{y}}_{i}\right)}^{2}/\Sigma {\left(y_{i}-{\bar{y}}_{i}\right)}^{2}$$

Linear relationships were described using Pearson’s correlation coefficient. To compare V at 90 and 95 wt.% recycled material, Welch’s test was used as a parametric comparison of means without assuming equal variances, and the Mann–Whitney test was used as a nonparametric analysis; Cohen’s d was also calculated. Because within-condition variability was very small under some conditions, the standardized effect size was interpreted cautiously and together with the direct difference in V and the distribution of observations. For the explicit models, cluster-robust standard errors were reported using 16 clusters corresponding to combinations of recycled-material level and Mn variant. Given the limited number of clusters, inference based on asymptotic standard errors was treated as supplementary; coefficient *p*-values were not used as the basis for model selection or for the main conclusions of the study. Uncertainty in differences between models was assessed using block bootstrap, with the entire recycled-material level as the resampling unit. Each bootstrap sample contained eight levels sampled with replacement together with all observations belonging to each sampled level. For key comparisons, 20,000 replicates were generated, and the mean RMSE difference, percentile 95% confidence interval (CI), and the proportion of replicates in which the candidate model had a lower error were reported.

### 2.10. Explainable Artificial Intelligence

XAI analysis was performed for models selected on the basis of outer-grouped LOSO cross-validation. Its purpose was not to reassess predictive accuracy, but to interpret the structure of the final models fitted to the complete set of 160 observations. For architecture B, the final second-order model based on Scrap, Mn treatment, and Fe was explained, whereas for architecture E the final Polynomial Ridge model using Scrap, Mn treatment, and *SF*_*Cr*0_ was explained. Exact Shapley values were calculated for the three original predictors using an empirical background comprising 160 observations. Global importance was defined as mean |SHAP| and normalized to 100%, with explanation additivity verified. Because Scrap and Fe were strongly interdependent, individual SHAP contributions were not interpreted as independent physical effects. Grouped Shapley was also used: in model B, the [Scrap + Fe] block was compared with Mn treatment, whereas in model E, Scrap was compared with the [*SF*_*Cr*0_ + Mn treatment] block. Because the groupings addressed different interpretive questions, their percentage values were not compared directly across models. All attributions were interpreted as model-specific predictive contributions rather than causal shares of metallurgical mechanisms. Generalization assessment remained based exclusively on OOF predictions from LOSO validation.

Calculations were performed in Python 3.13.5. The models were implemented using scikit-learn 1.8.0, XGBoost 3.1.3, and CatBoost 1.2.8. XAI analysis was performed using SHAP 0.50.0; statistical calculations were supported by NumPy 2.3.5, SciPy 1.17.0, and statsmodels 0.14.6. For stochastic algorithms, random_state = 42 was used when the implementation provided this parameter; this setting was used to ensure reproducibility and was not treated as a tuned hyperparameter. Group bootstrap was generated using a fixed random-generator seed. Preprocessing, tuning, and outer validation were carried out programmatically, with strict separation of training and test data.

## 3. Results

### 3.1. Effect of Recycled Material Content and Mn Correction on Porosity

**Figure 1:**
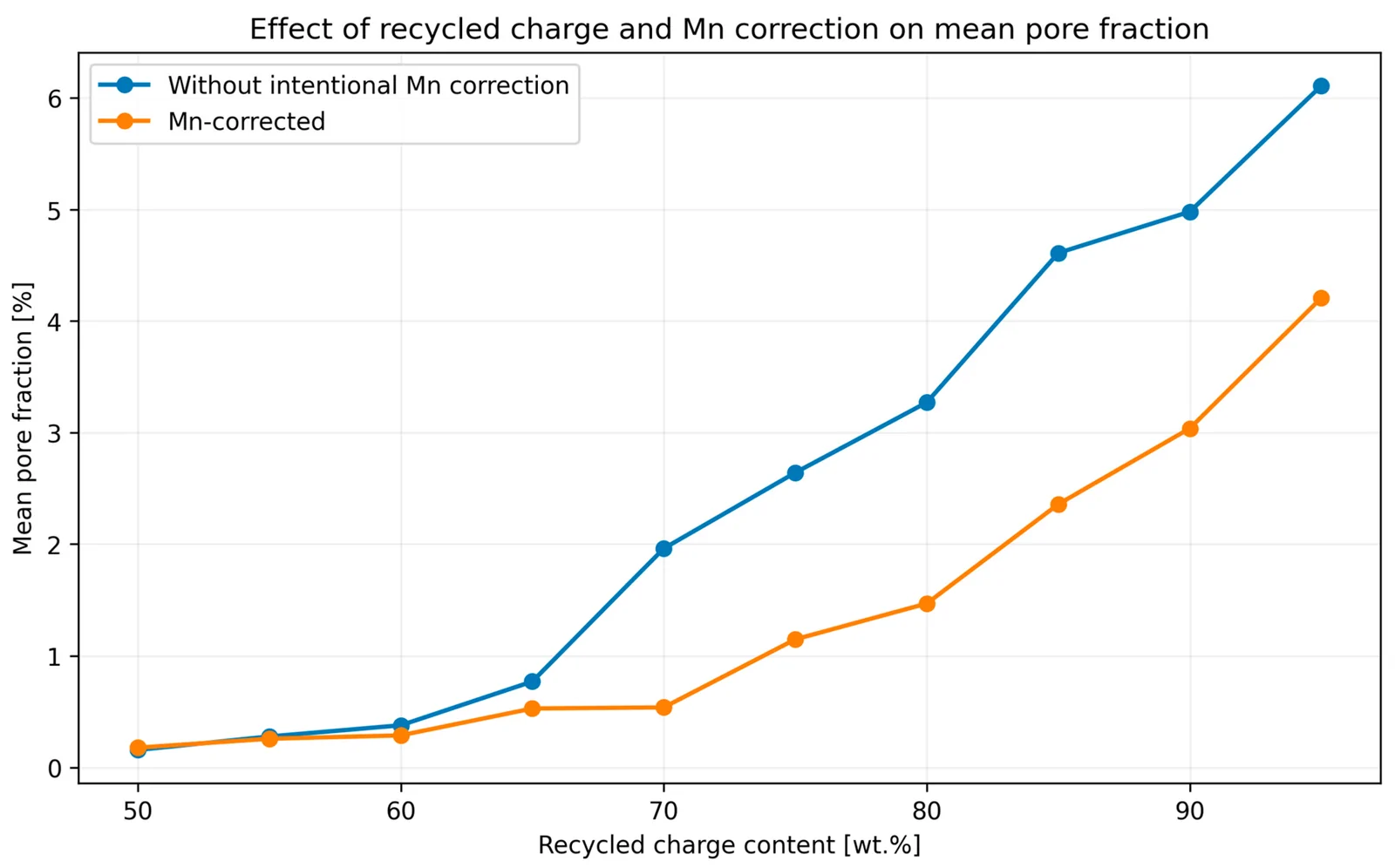
Mean porosity area fraction as a function of recycled material content for both Mn variants.

The mean porosity area fraction increased with increasing recycled charge content in the series without intentional Mn correction. It rose from 0.160% at 50 wt.% scrap to 6.111% at 95 wt.% scrap, i.e., by approximately 38-fold. The trend was nonlinear: values remained below 1% up to 65 wt.% scrap, whereas a markedly stronger increase was observed from 70 wt.% onward. The complete condition means are listed in Table 8, and the trend is shown in Figure 1. Mn correction reduced the mean porosity area fraction over almost the entire investigated range. At 60 wt.% scrap, porosity decreased from 0.380 to 0.290%, and at 70 wt.% from 1.961 to 0.540%. At 95 wt.%, the corresponding values were 6.111% without correction and 4.208% after Mn correction. The relative effect of correction was not constant. At 50 wt.% scrap, porosity increased slightly from 0.160 to 0.180%. The largest relative reduction, approximately 72.5%, was obtained at 70 wt.% scrap. At higher contents, the relative reduction gradually decreased, reaching approximately 31.1% at 95 wt.%.

Representative light-microscopy images document the investigated conditions across increasing recycled-material contents and enable qualitative comparison of the non-corrected and Mn-corrected variants at 90 and 95 wt.% recycled material. These images complement the quantitative porosity measurements. Additional SEM observations at 70, 80, and 90 wt.% recycled material provide higher-resolution morphological context.

### 3.2. Evolution of Fe, Mn, and the Reduced Sludge Factor

**Figure 2:**
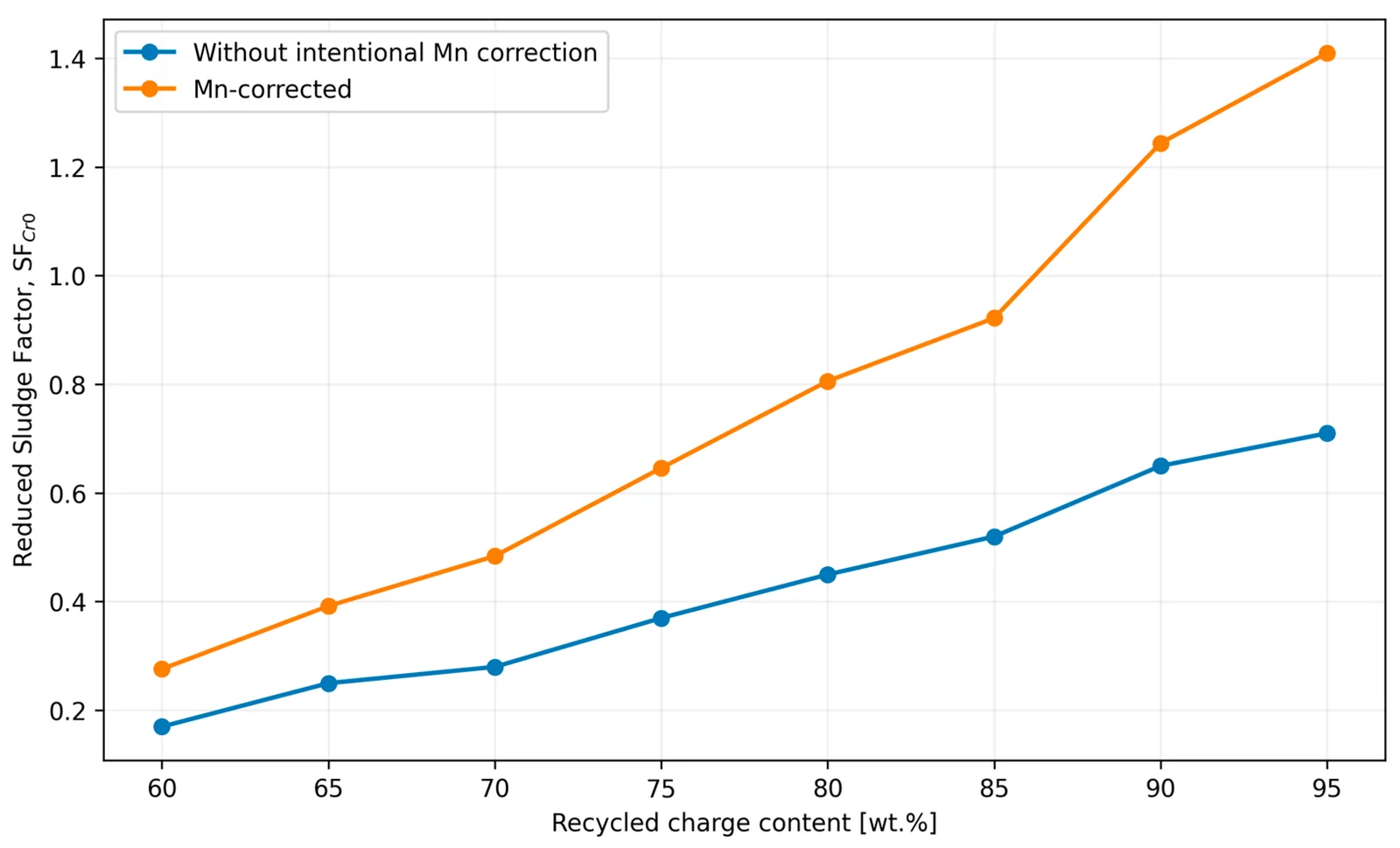
Change in the reduced Sludge Factor *SF*_*Cr*0_ with increasing recycled material content.

Over the range of 60–95 wt.%, increasing scrap content was accompanied by a systematic increase in Fe content. In the series without intentional Mn correction, mean Fe increased from 0.1299 wt.% at 60 wt.% to 0.6899 wt.% at 95 wt.%. In the Mn-corrected series, Fe followed a similar trend, whereas Mn was increased toward Mn/Fe ≈ 0.5. Changes in Fe, Mn, and *SF*_*Cr*0_ are summarized in Table 9 and shown in Figure 2. Consequently, *SF*_*Cr*0_ = (1 × %Fe) + (2 × %Mn) increased in both series. Without correction, its mean value increased from 0.1697 to 0.7097, whereas after Mn correction it increased from 0.2757 to 1.4097.

For all 160 paired observations, the correlation between *SF*_*Cr*0_ and mean porosity area fraction was r = 0.476. After separating the Mn variants, the correlation was much stronger: r = 0.983 without correction and r = 0.982 after correction. The global *SF*_*Cr*0_–porosity relationship is therefore strongly conditioned by the experimental structure.

### 3.3. Incremental Value of Chemical Information in Transparent Models

**Figure 3:**
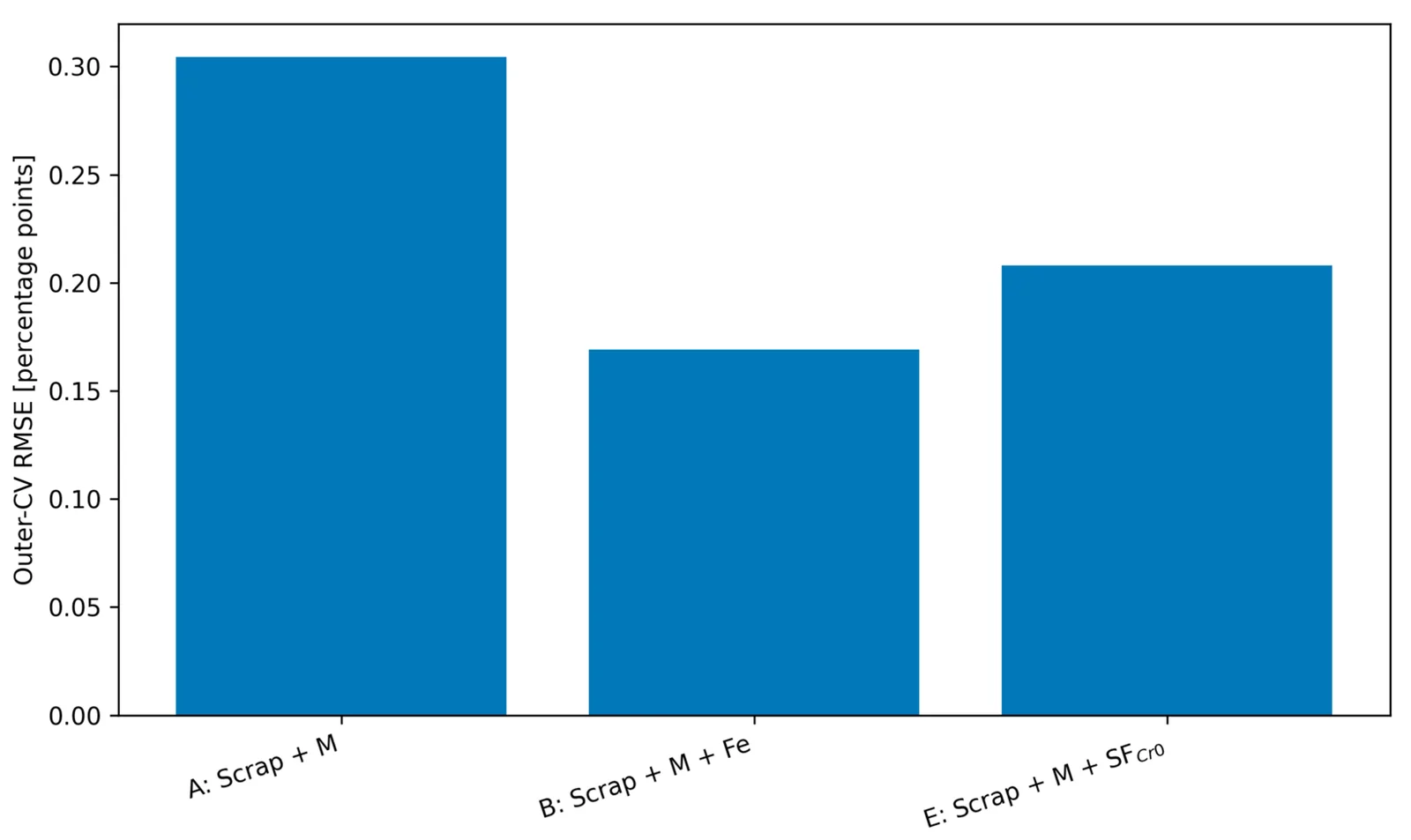
RMSE of explicit models A, B, and E in outer-grouped LOSO cross-validation.

Before comparing the full set of algorithm families, a controlled analysis of three explicit models with the same general functional structure was performed. Model A represented process information comprising recycled material content and the Mn-correction variant; model B extended this representation by adding sample-specific Fe content, whereas model E added the reduced descriptor *SF*_*Cr*0_. All three models were evaluated using the same outer-grouped LOSO cross-validation. The controlled A–B–E comparison is summarized in Table 10 and Figure 3.

Adding sample-specific Fe reduced RMSE relative to model A by approximately 44.5%, whereas using *SF*_*Cr*0_ reduced RMSE by approximately 31.7%. Block bootstrap by recycled-material level confirmed the stability of both improvements: for A → B, mean ΔRMSE was 0.1346 percentage points (p.p.) (95% CI 0.0706–0.2005), and for A → E it was 0.0977 p.p. (95% CI 0.0617–0.1379). The direct difference between models B and E was less clear: the mean RMSE difference was 0.0371 p.p., but the 95% CI included zero (−0.0294 to 0.1045). Fe therefore produced a lower nominal error for mean porosity, but the available data do not support treating this advantage over *SF*_*Cr*0_ as being as stable as the advantages of both chemical representations over benchmark A.

In the subsequent ranking of architectures, the best algorithm for each feature representation is reported. Therefore, the value for model A in the next section (best Kernel Ridge RBF, RMSE = 0.2890) differs from that of the explicit model A used above for the controlled comparison of incremental feature value (RMSE = 0.3045).

#### Condition-Level Sensitivity Analysis

Because the 160 chemistry–porosity observations were nested within 16 experimental conditions, an additional condition-level sensitivity analysis was performed to determine whether the predictive performance of architecture B depended on the use of multiple independently produced castings within each condition. The 10 castings belonging to each combination of recycled-material level and Mn-treatment state were aggregated to their condition means, resulting in 16 observations (eight recycled-material levels × two Mn variants). Mean Fe content and MeanPor were calculated independently for each condition.

The same second-order structure used for model B was then evaluated by Leave-One-Scrap-Level-Out cross-validation. In each iteration, both condition means corresponding to one recycled-material level were excluded from model fitting. The condition-level model achieved R^2^ = 0.99100, RMSE = 0.16864 percentage points, and MAE = 0.15088 percentage points. These values were nearly identical to those obtained in the primary 160-casting analysis (R^2^ = 0.9910, RMSE = 0.1691, MAE = 0.1508).

This sensitivity analysis indicates that the high predictive performance was not created by treating the 10 castings within each condition as independent recycled-material levels. The dominant predictive structure was retained after aggregation to the 16 experimental condition means. Nevertheless, the chemistry-resolved experiment contained only eight distinct recycled-material levels, and this analysis does not provide independent external validation. Generalization beyond the investigated process window therefore requires validation using independent production campaigns and/or manufacturing conditions.

### 3.4. Comparison of Predictor Architectures

**Figure 4:**
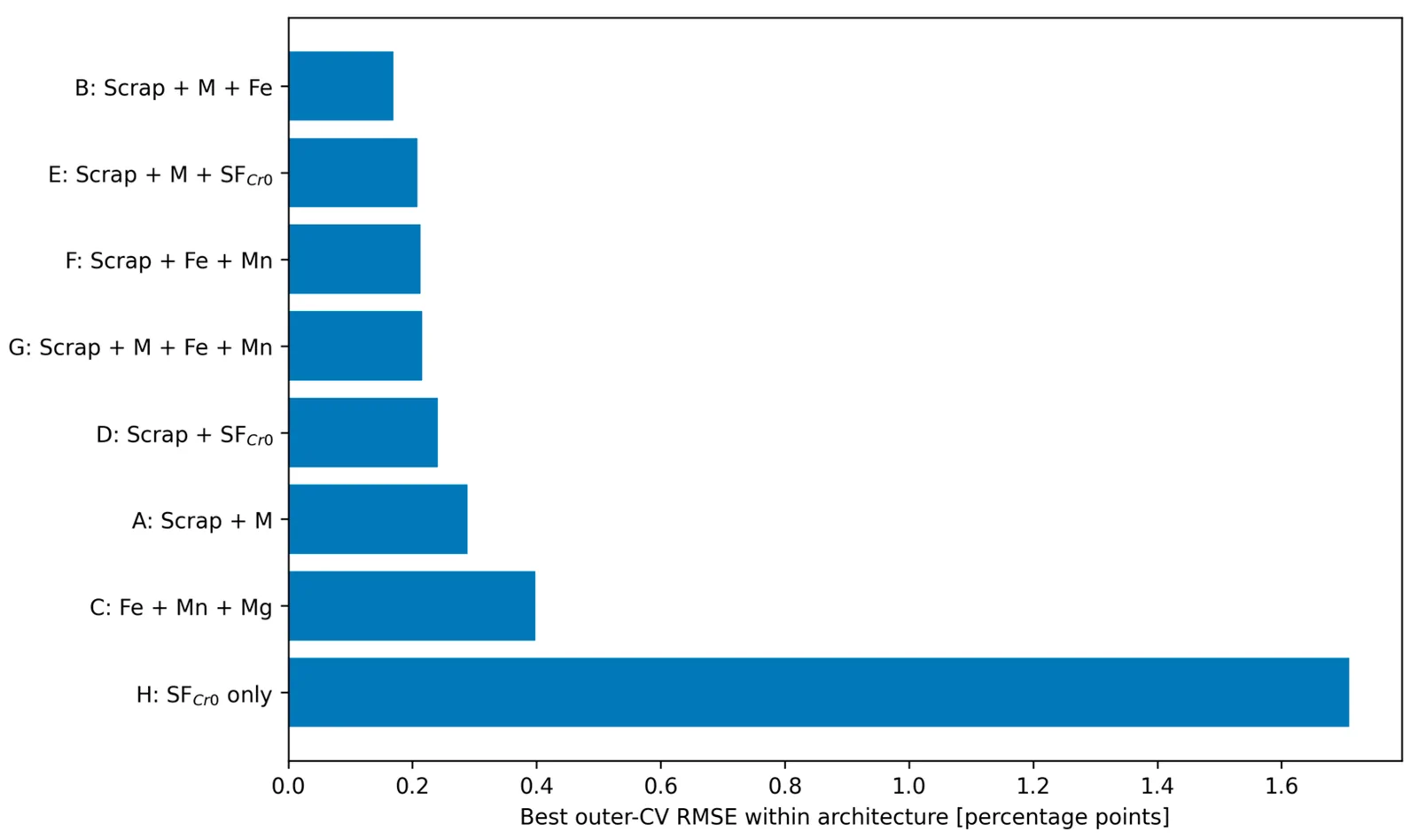
Lowest RMSE achieved for each input architecture.

For each of the eight architectures, the model family achieving the lowest RMSE in outer-grouped LOSO cross-validation was identified (Figure 4). These results should be distinguished from the controlled comparison of explicit A–B–E models presented in Section 3.3, because the position of an architecture in Table 11 depends jointly on the feature representation and the algorithm family. The lowest error was obtained by architecture B (Scrap + Mn treatment + Fe), for which second-order regression with predefined interactions achieved R^2^ = 0.9910 and RMSE = 0.1691. The second-lowest RMSE was obtained by architecture E with *SF*_*Cr*0_ (R^2^ = 0.9863; RMSE = 0.2080).

### 3.5. Predictive Performance Across Model Families

**Figure 5:**
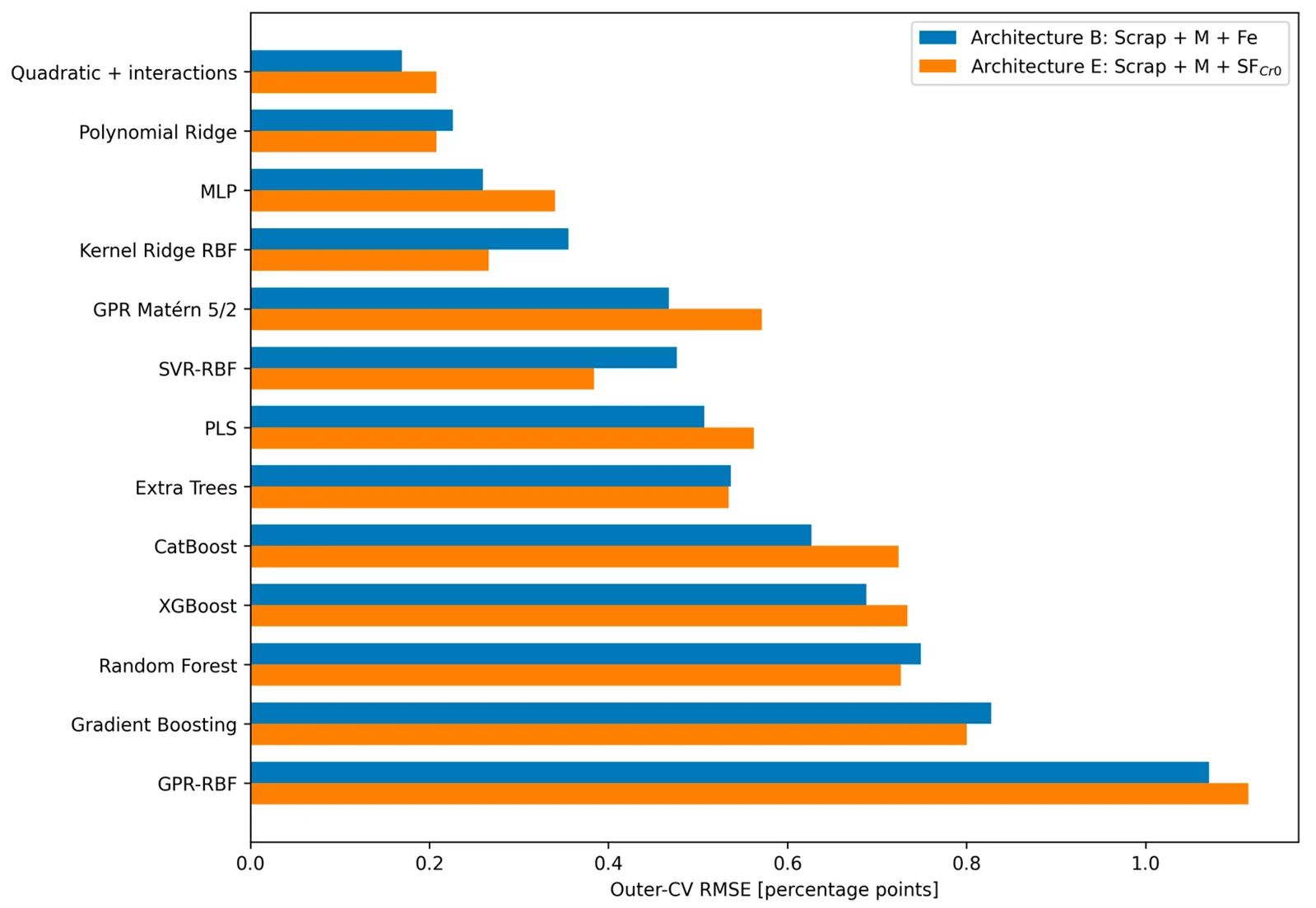
RMSE of 13 model families for architectures B and E.

Architectures B and E were compared across 13 model families (Figure 5). In B, the lowest RMSE was achieved by the transparent second-order regression with predefined interactions (RMSE = 0.1691), followed by Polynomial Ridge (0.2261) and MLP (0.2597). In E, the two best models were practically equivalent: Polynomial Ridge achieved RMSE = 0.20802, while the explicit second-order model achieved 0.20809. Within the investigated dataset, more complex models did not show a systematic advantage. B had a lower RMSE in 7 of the 13 families and E in 6 of 13 (Table 12); however, this count is purely descriptive and does not constitute a formal test of the superiority of the Fe representation over *SF*_*Cr*0_.

Bootstrap resampling of the entire recycled-material levels indicated a consistently lower RMSE for the explicit model B than for Polynomial Ridge and MLP within the same feature architecture: the mean differences were approximately 0.0565 p.p. (95% CI 0.0184–0.0956) and 0.0905 p.p. (95% CI 0.0406–0.1458), respectively.

### 3.6. Final Models and OOF Error Behavior

**Figure 6:**
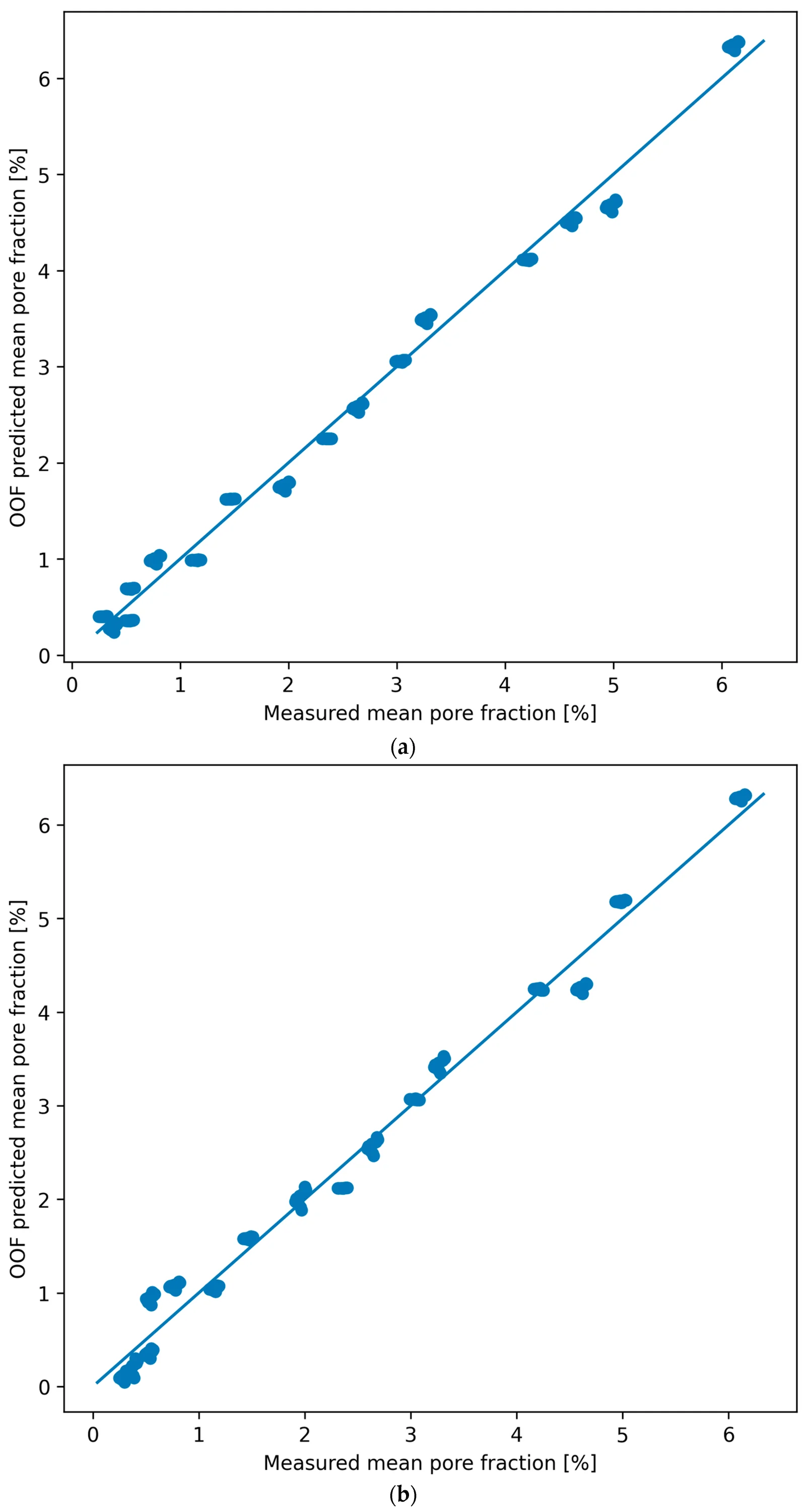

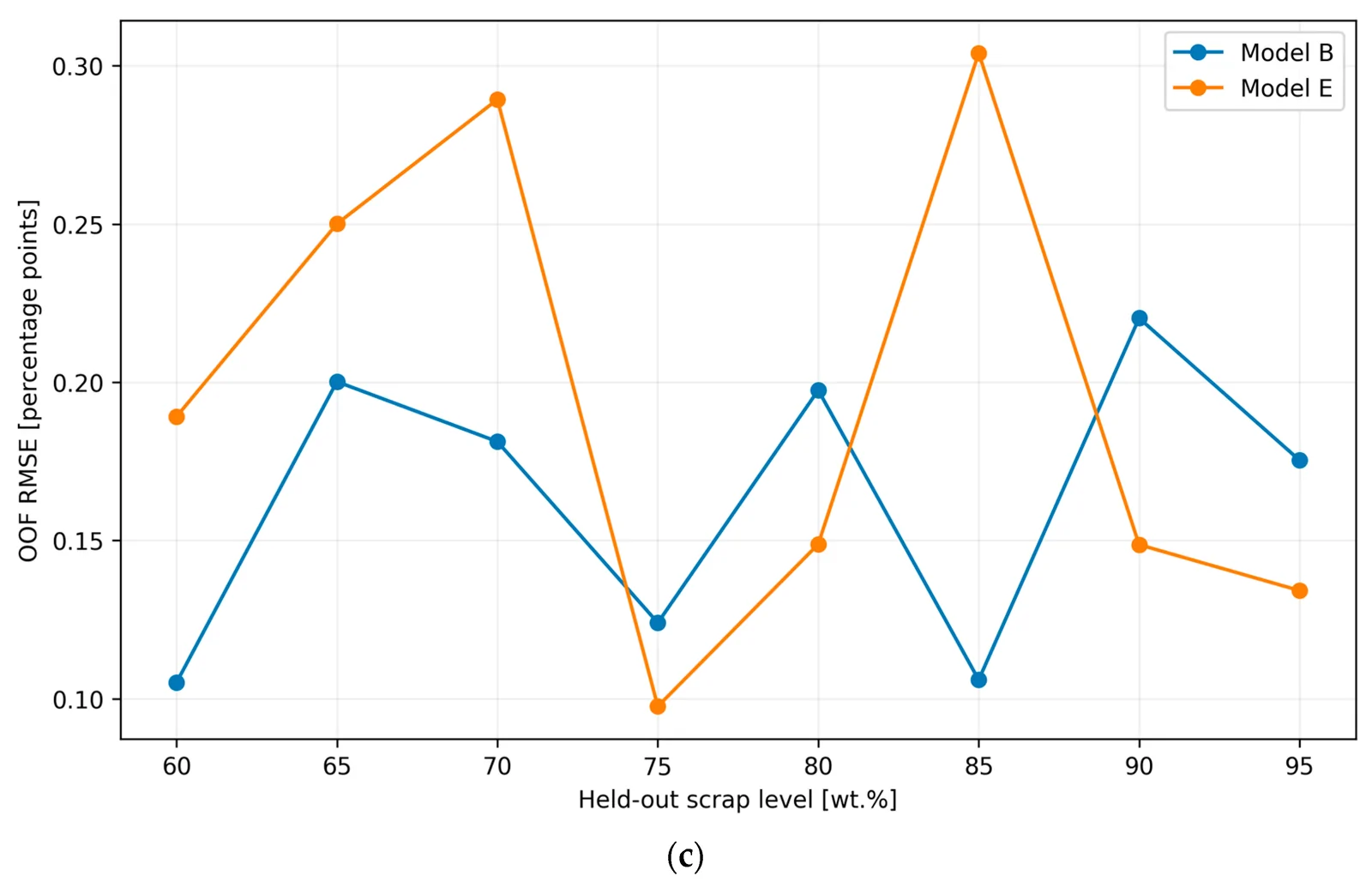
(**a**) Out-of-fold (OOF) predictions of the final model B versus measured values. (**b**) Out-of-fold (OOF) predictions of the best model E (Polynomial Ridge) versus measured values. (**c**) RMSE of models B and E in individual outer folds of LOSO validation.

After fitting to the complete dataset, the final model B had the form shown in Equation (6). The model coefficients are listed in Table 13, and agreement between OOF predictions and measured values is shown in Figure 6a.

(6) $$\begin{array}{r} \hat{P}=-0.89954288-0.15592265S+12.93916774M-12.79157332\mathrm{Fe}+\\ 0.0034056152S^{2}-0.24027597(S\times M)+11.38744311(\mathrm{Fe}\times M) \end{array}$$

Only OOF predictions were used as measures of model performance: R^2^ = 0.9910, RMSE = 0.1691, and MAE = 0.1508. In Equation (6), $\hat{P}$ denotes the predicted mean porosity area fraction [%], S is the recycled material content [wt.%], M is the binary Mn-correction variant (M = 0: without intentional correction; M = 1: after correction), and Fe is the sample-specific Fe content [wt.%]. The equation was developed and validated for S = 60–95 wt.% and for the chemical-composition range represented by the investigated dataset; it should not be used for extrapolation beyond this domain without independent validation.

The signs of individual coefficients are conditional model parameters and should not be interpreted in isolation as independent physical effects. The model contains a quadratic term and interactions, and Scrap and Fe are strongly interdependent; therefore, the effect of changing a single predictor depends on the values of the remaining variables. In particular, the negative coefficient for Fe does not mean that increasing Fe physically reduces porosity. The coefficient *p*-values in Table 13 were treated as supplementary only because of the limited number of clusters.

The best predictive model for architecture E was Polynomial Ridge. Because the explicit second-order model with the same architecture achieved an almost identical RMSE, its equation is presented only as a transparent comparison model. The coefficients in Equation (7), listed in Table 14, are not the coefficients of the Polynomial Ridge model, whereas the predictions shown in Figure 6b come from the actual best Polynomial Ridge model.

(7) $$\begin{array}{r} \hat{P}=-5.17689020-0.01332365S+13.89000087M-11.23010841\;{\mathrm{SF}}_{\mathrm{Cr}0}\\ +0.0022924278S^{2}-0.27005925(S\times M)+12.36555095({\mathrm{SF}}_{\mathrm{Cr}0}\times M) \end{array}$$

The coefficient *p*-values of the explicit model E, as in model B, are supplementary and do not provide a basis for inference about physical mechanisms.

The advantage of B was not present at every recycled-material level. B had a lower RMSE at 60, 65, 70, and 85 wt.%, whereas E had a lower RMSE at 75, 80, 90, and 95 wt.% (Table 15). The fold-specific results therefore show that the lower global RMSE of model B did not arise from uniform superiority at every unseen level; architecture E achieved a lower error in four of the eight outer folds, including at 90 and 95 wt.% recycled material.

For B, 90% of absolute OOF errors were ≤0.2620 p.p., 95% were ≤0.2749 p.p., and the maximum was 0.3832 p.p. For E, the corresponding values were 0.3417, 0.3988, and 0.4474 p.p.

### 3.7. Predictive Value of Individual Additional Chemical Descriptors

**Figure 7:**
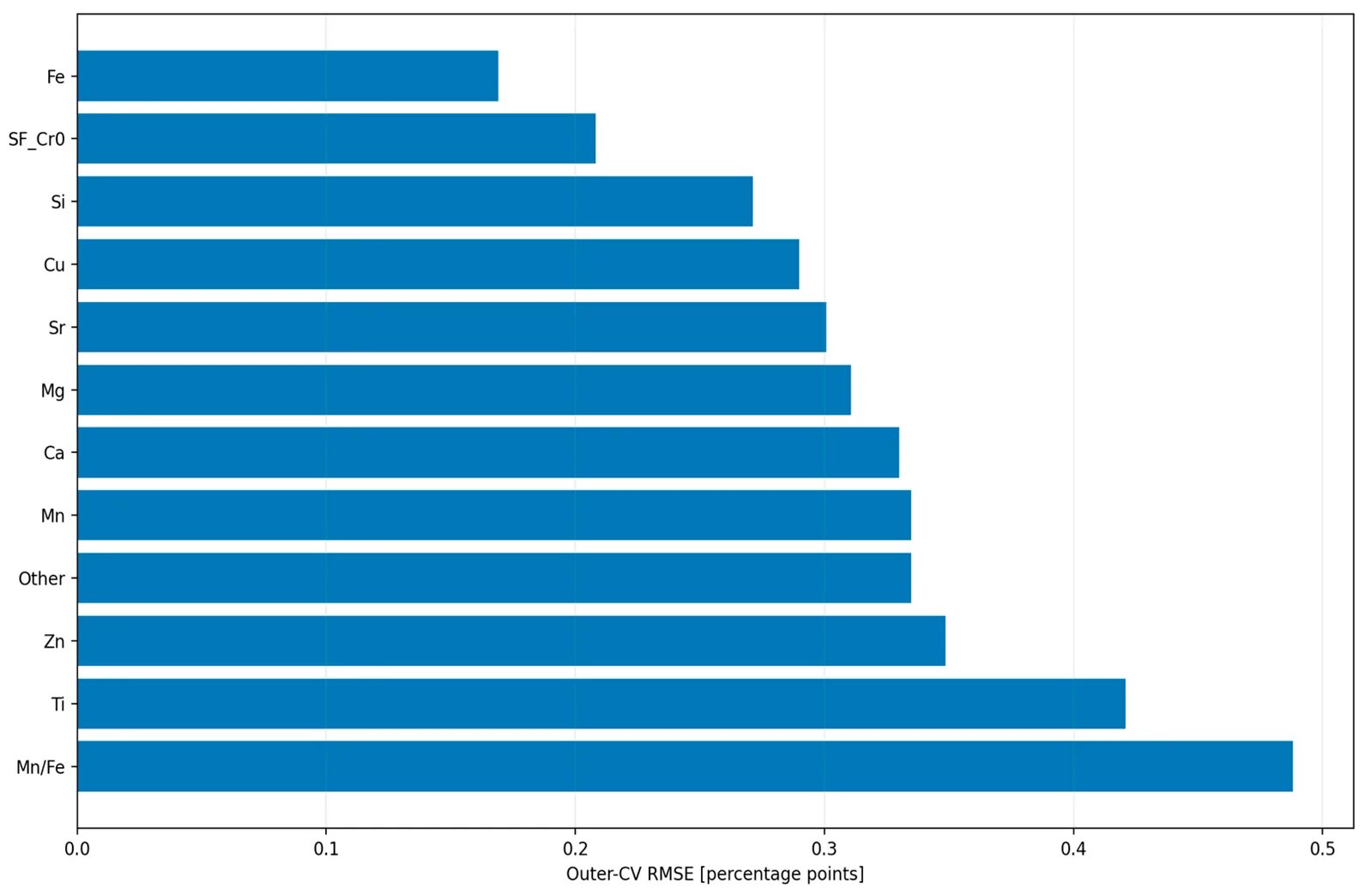
RMSE comparison of individual additional chemical descriptors using an identical model structure.

Each available chemical descriptor X was introduced into the same structure S + M + X + S^2^ + S × M + X × M. This allowed its incremental predictive value to be compared without changing the model class. The full comparison is presented in Table 16 and Figure 7.

Fe provided the greatest incremental predictive value among the evaluated individual descriptors for MeanPor (RMSE = 0.1691), while *SF*_*Cr*0_ achieved the second-lowest nominal RMSE (0.2081). Bootstrap analysis indicated a consistently lower error for Fe relative to Si and for *SF*_*Cr*0_ relative to Si. The direct Fe–*SF*_*Cr*0_ difference remained nominal because the 95% CI of the difference included zero (Table 17).

### 3.8. Prediction of Additional Porosity Descriptors

**Figure 8:**
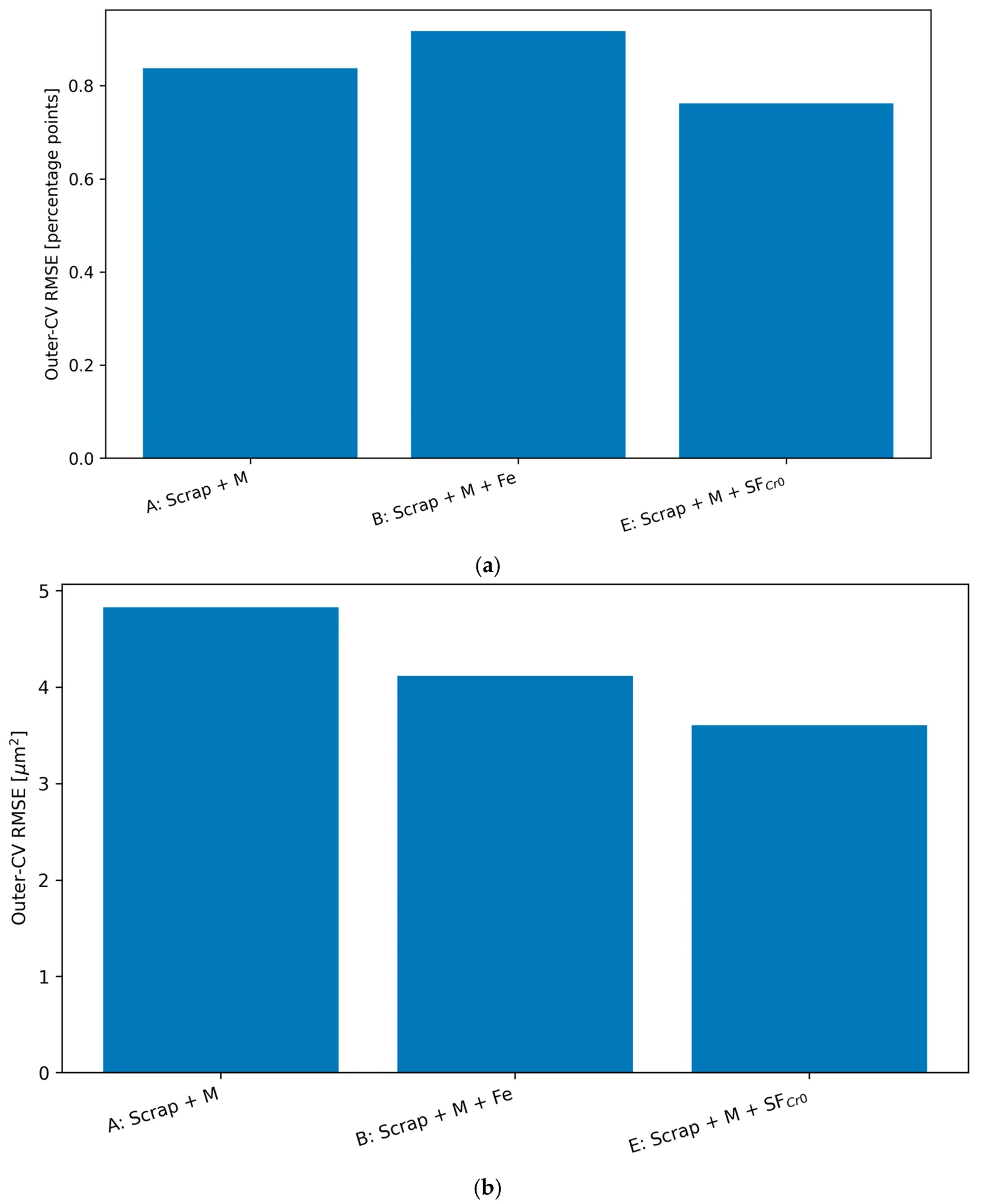
(**a**) RMSE of architectures A, B, and E for MostPor. (**b**) RMSE of architectures A, B, and E for mean pore area.

The explicit architectures were also compared for MostPor and PoreArea. For both additional targets, architecture E with *SF*_*Cr*0_ achieved the lowest nominal RMSE, although the strength of evidence differed between responses: for MostPor, the advantage of E over B was stable in the bootstrap, whereas for PoreArea the bootstrap intervals for the E–B and E–A comparisons included zero. The comparison of the three targets is presented in Table 18, and the key results are illustrated in Figure 8a,b. To maintain comparability across targets, the same class of explicit models was used; therefore, the values in Table 18 do not necessarily correspond to the best algorithmic results in Table 11.

For MostPor, model E achieved R^2^ = 0.9511 and RMSE = 0.7624, whereas B achieved R^2^ = 0.9292 and RMSE = 0.9171. Bootstrap analysis indicated a consistently lower RMSE for E relative to B for this target: the mean improvement was 0.1536 p.p., with a 95% CI of 0.0546–0.2538. For PoreArea, E also achieved the nominally lowest RMSE (3.6033 µm^2^), but the bootstrap intervals for the E–B and E–A comparisons included zero. The corresponding bootstrap comparisons are summarized in Table 19.

### 3.9. Explainable Artificial Intelligence

XAI analysis was performed for the final models fitted to the complete dataset of 160 observations: model B and the best model of architecture E (Polynomial Ridge). The purpose of SHAP was to interpret the structure of information used by the final models rather than to reassess their generalization ability, which remained based on OOF predictions from LOSO validation. Global importance was defined as mean |SHAP| and normalized to 100%. The corresponding global SHAP importance values for models B and E are presented in Table 20.

In B, Scrap accounted for 58.14% of global importance, Fe for 26.28%, and Mn treatment for 15.57%. In E, the corresponding shares were 64.87% for Scrap, 32.49% for *SF*_*Cr*0_, and 2.64% for Mn treatment. The small additional contribution of the binary Mn variable in E is consistent with the fact that *SF*_*Cr*0_ already contains information on the actual Mn content.

**Figure 9:**
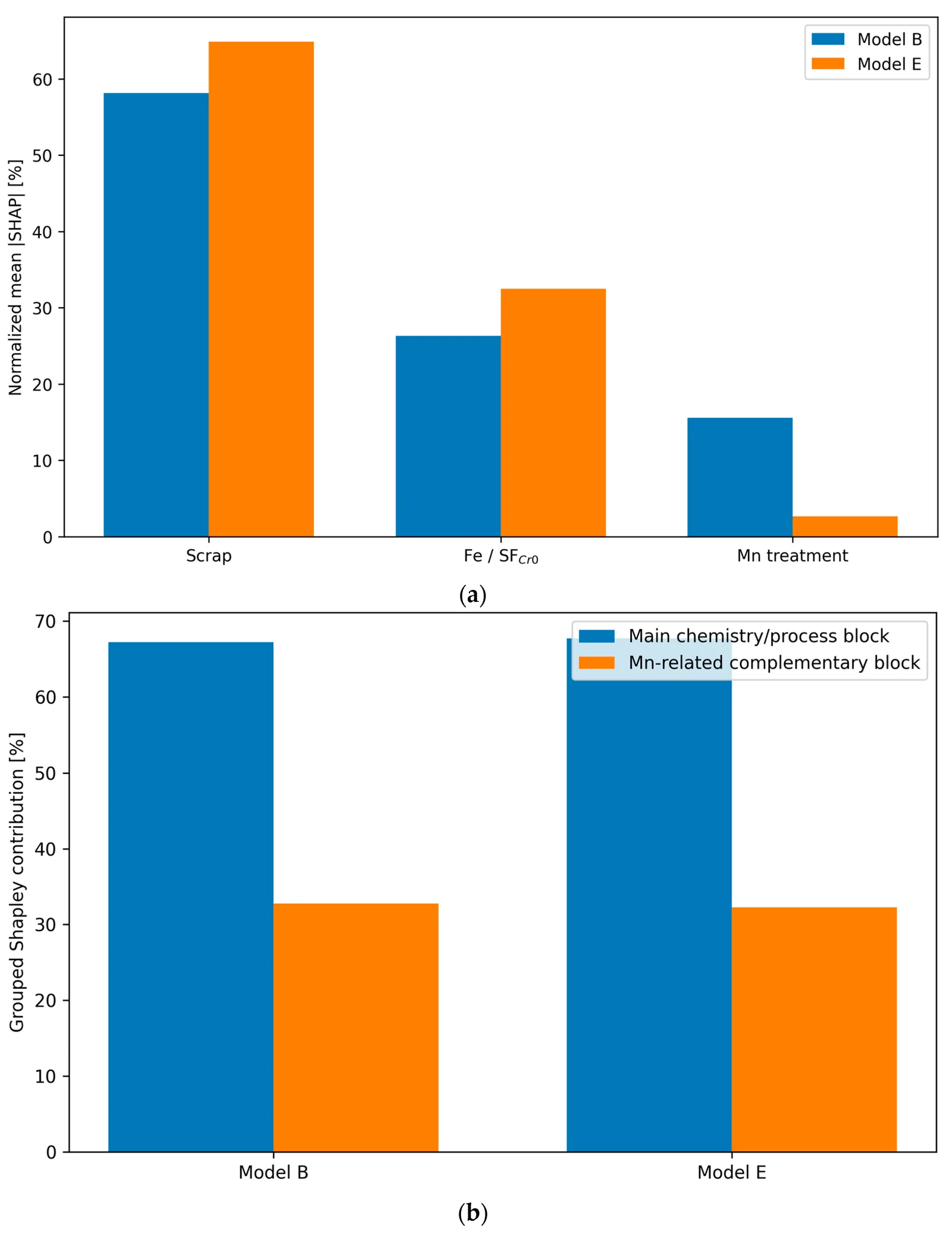
(**a**) Normalized global mean |SHAP| values for models B and E. (**b**). Grouped Shapley values. For B: [Scrap + Fe] vs. Mn treatment; for E: Scrap vs. [*SF*_*Cr*0_ + Mn treatment]. The groupings address different interpretive questions and should not be compared directly percentage-for-percentage.

Because of the strong Scrap–Fe dependence, grouped Shapley was also performed. In B, the Scrap + Fe block accounted for 67.24%, whereas Mn treatment accounted for 32.76%. In E, Scrap accounted for 67.74%, while the *SF*_*Cr*0_ + Mn treatment block accounted for 32.26%. Because the grouping scheme differed between models B and E, the percentage values of the two analyses were not compared directly. In model B, grouping reduced the arbitrary splitting of attribution between the interdependent Scrap and Fe variables, whereas in E, *SF*_*Cr*0_ was grouped with the binary Mn-correction indicator because of the shared information related to Mn. The normalized global SHAP importance values and the corresponding grouped Shapley attributions are illustrated in Figure 9a,b. The grouped Shapley values are also summarized in Table 21.

SHAP values describe the allocation of predictive information within a given fitted model. They should not be interpreted as the percentage contribution of physical mechanisms or as evidence of causality; for example, the 58.14% share attributed to Scrap does not mean that 58.14% of the physical mechanism of porosity formation is caused by recycled material content.

### 3.10. Pore Morphology Described by Shape Factor V

The V factor was analyzed separately from *SF*_*Cr*0_. The data summarized in Table 22 comprised 60 observations without intentional Mn correction for 70–95 wt.% recycled material and 20 observations after Mn correction only at 90 and 95 wt.%. Because the experimental plan was covered incompletely and asymmetrically, the V analysis was treated as supplementary, and direct comparisons of the effect of Mn correction were performed only at 90 and 95 wt.%, i.e., the levels for which both variants were available.

All available observations from the series without intentional Mn correction had V < 0.8. Under the two conditions for which a direct comparison after Mn correction was available (90 and 95 wt.%), all observations reached V ≥ 0.8. However, the value 0.8 should be treated solely as an auxiliary morphological criterion used for the investigated system, not as a universal boundary for classifying the pore-formation mechanism.

**Figure 10:**
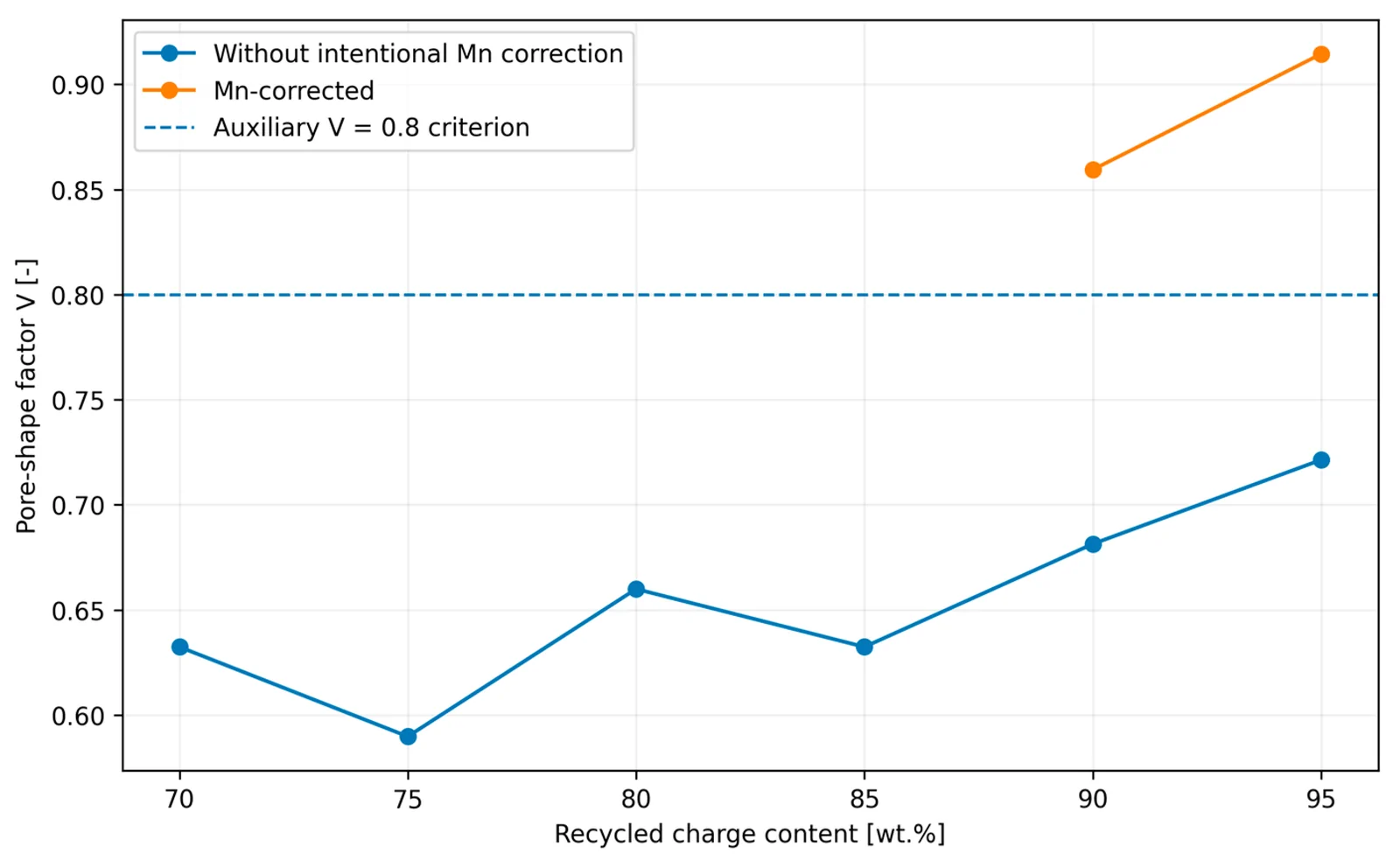
Pore shape factor V as a function of recycled material content. The V = 0.8 line denotes an auxiliary morphological criterion rather than a universal boundary for the pore-formation mechanism.

A high condition-level mean V does not imply that every individual pore cross-section is regular or rounded. The representative LM fields still contain isolated irregular defects after Mn correction; the observed increase in V is therefore interpreted as a shift in the average pore cross-sectional geometry rather than complete elimination of irregular pores. The variation in V with recycled material content, together with the auxiliary V = 0.8 reference line, is illustrated in Figure 10. The statistical comparison of V between the Mn variants at 90 and 95 wt.% recycled material is summarized in Table 23.

Because of the very small within-condition variance, the standardized effect size of Cohen’s d reached extremely high values and was treated as supplementary; greater emphasis was placed on the direct difference in V and the complete separation of the observed ranges. The nominal correlation between V and *SF*_*Cr*0_ in the entire unbalanced 80-observation dataset was r = 0.979, but because the recycled-material levels were covered differently between the Mn variants, this value was not interpreted as a measure of an independent physical relationship. The global correlation between V and MeanPor was weak (r = 0.186), consistent with the interpretation that porosity amount and pore cross-sectional geometry describe partly different aspects of the defect. The results indicate more regular pore cross-sectional geometry after Mn correction at 90 and 95 wt.%, together with lower MeanPor. However, V analysis and 2D LM images alone do not allow the pore-formation mechanism to be determined unambiguously or support a conclusion that shrinkage porosity was converted into gas porosity. The retained summary results do not include the original pore-by-pore object lists or the complete casting-level PoreArea distributions. Therefore, new pore-object counts, pore-size quartiles, and object-level distributional comparisons are not reported. The original V and PoreArea summary results are retained as outcomes of the completed analyses.

### 3.11. Representative LM Images Supporting the Observed Porosity Trends

**Figure 11:**
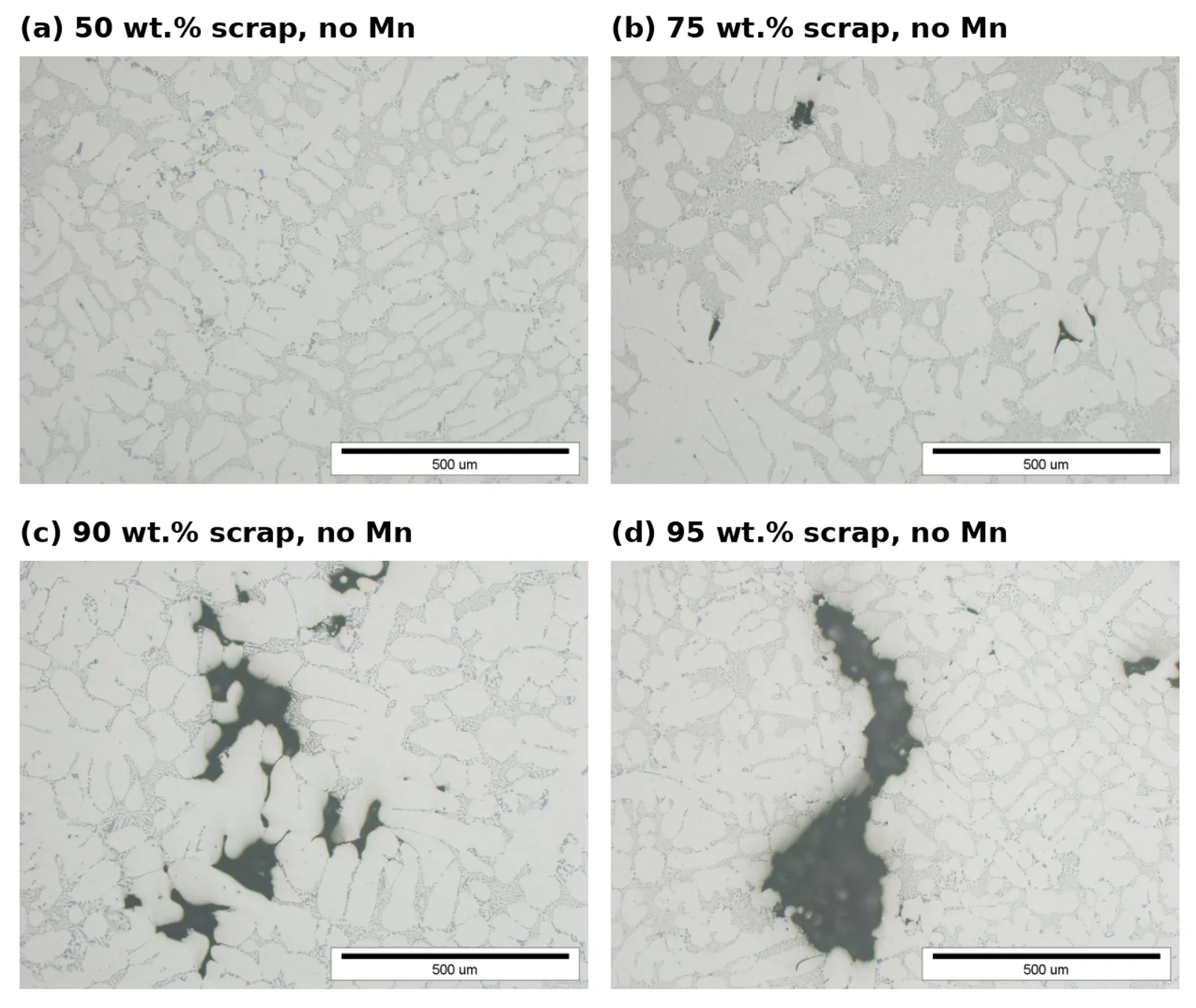
Representative LM images of low-pressure-cast AlSi7Mg alloy without intentional Mn correction: (**a**) 50 wt.% recycled material (Fe data unavailable); (**b**) 75 wt.%, 0.31 wt.% Fe; (**c**) 90 wt.%, 0.61 wt.% Fe; and (**d**) 95 wt.%, 0.69 wt.% Fe. Panels were acquired at 100× magnification. The images show representative fields and provide a qualitative complement to the quantitative porosity measurements; they do not constitute an independent basis for identifying the composition of intermetallic phases.

Representative LM images selected from the experimental series are shown in Figure 11. Conditions corresponding to 50, 75, 90, and 95 wt.% recycled material without intentional Mn correction are presented. The images provide a qualitative complement to the quantitative porosity trend discussed in Section 3.1; they do not constitute an independent basis for identifying the composition of intermetallic phases.

**Figure 12:**
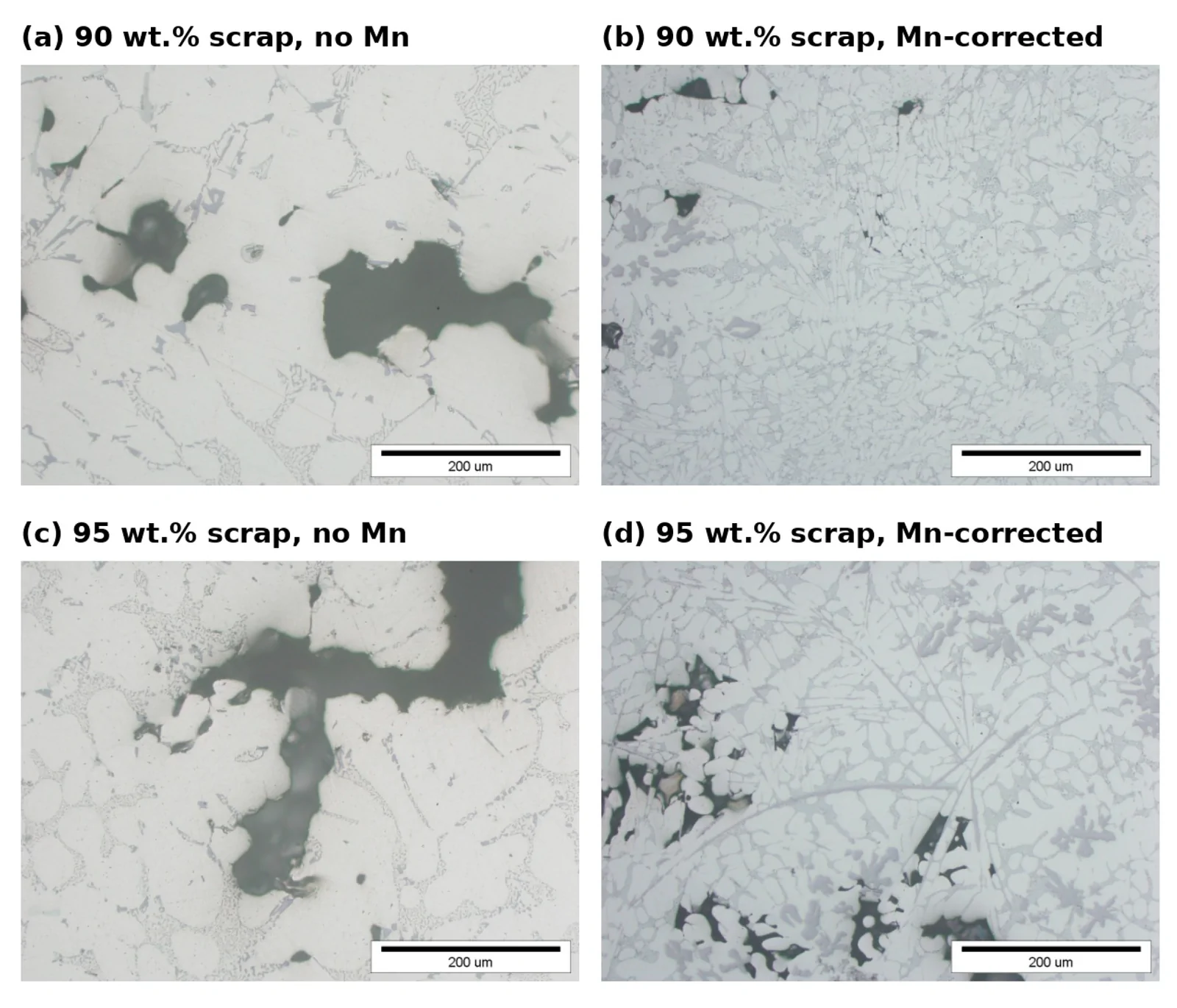
Representative LM images illustrating the effect of Mn correction at high recycled-material content: (**a**) 90 wt.% without intentional Mn correction, 0.61 wt.% Fe; (**b**) 90 wt.% after Mn correction, 0.61 wt.% Fe + 0.318 wt.% Mn; (**c**) 95 wt.% without intentional Mn correction, 0.69 wt.% Fe; and (**d**) 95 wt.% after Mn correction, 0.69 wt.% Fe + 0.363 wt.% Mn. All panels were acquired at 200× magnification. The images should be interpreted together with the quantitative porosity and V-factor results; they do not constitute an independent basis for identifying phases or the pore-formation mechanism.

The effect of Mn correction at the two highest recycled-material contents is illustrated in Figure 12. At both 90 and 95 wt.%, a different defect population and pore cross-sectional geometry are visible after Mn correction compared with the corresponding conditions without correction. These observations are consistent with lower MeanPor and higher V after Mn correction, but the images themselves were not used to classify the physical pore-formation mechanism or to unambiguously identify intermetallic phases.

### 3.12. Representative Otsu Segmentation

**Figure 13:**
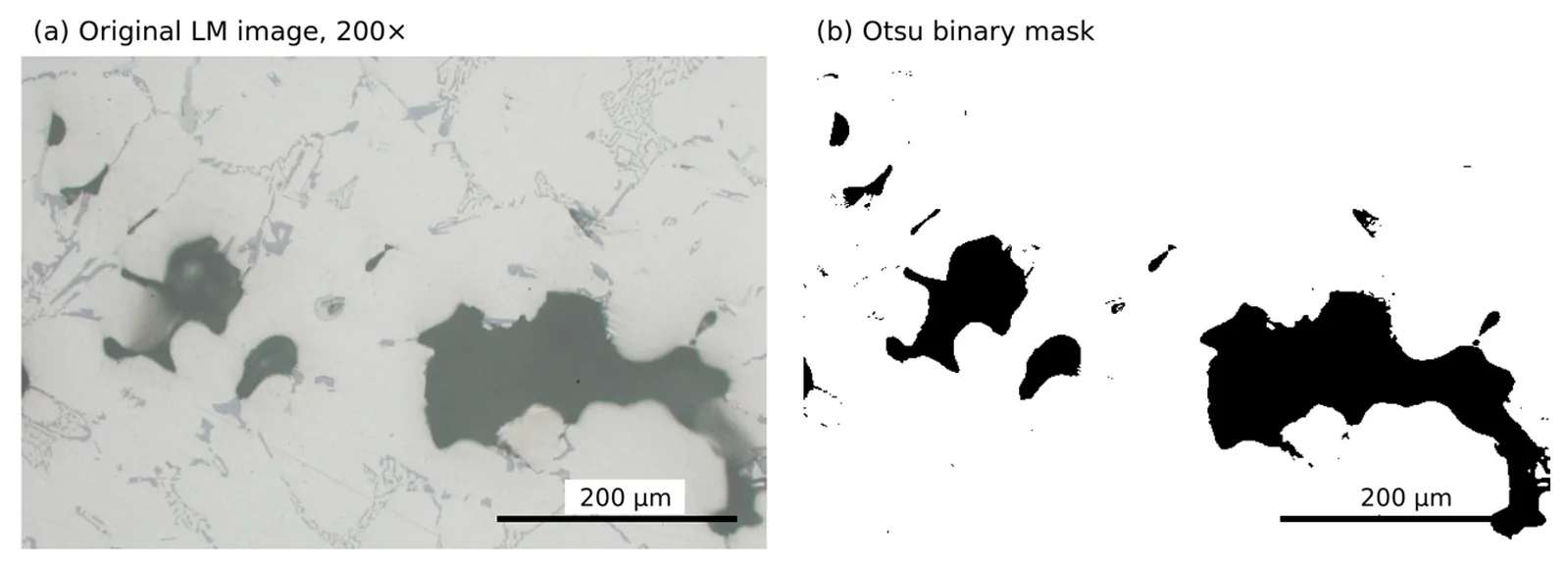
Representative original LM image for the 90 wt.% recycled-material condition without intentional Mn correction and the corresponding Otsu binary mask. Magnification: 200×; scale bar: 200 µm.

A representative 200× LM field and the corresponding binary mask obtained by automatic Otsu thresholding are shown in Figure 13. The example illustrates the image-to-mask step used in the quantitative porosity workflow; no manual threshold correction was applied.

### 3.13. Representative SEM Observations

**Figure 14:**
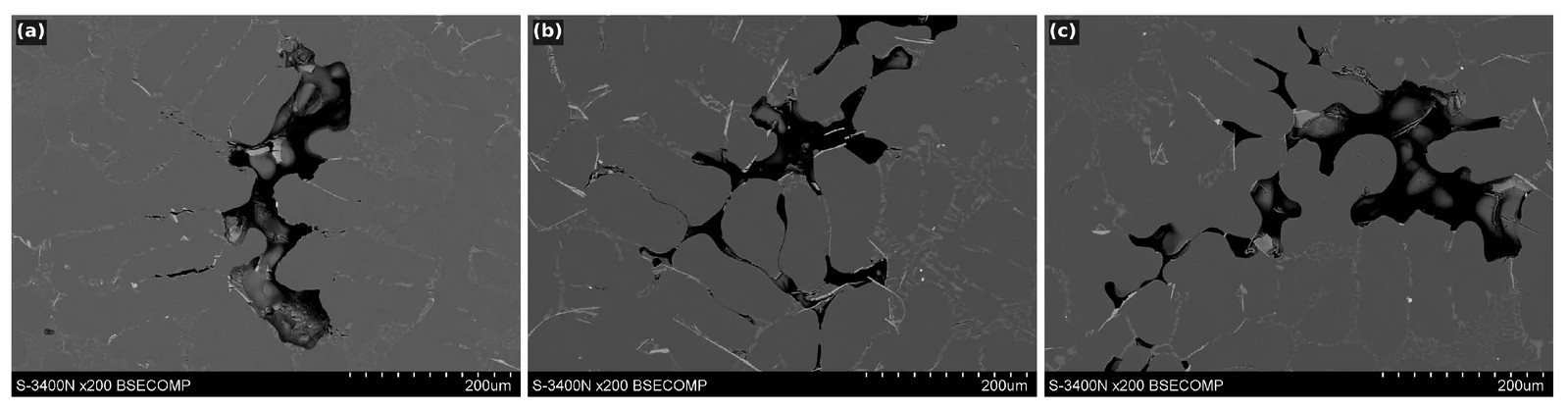
Representative SEM-BSE images of the investigated AlSi7Mg alloy at recycled-material contents of (**a**) 70 wt.%, (**b**) 80 wt.%, and (**c**) 90 wt.%. Hitachi S-3400N, 200×; scale bar: 200 µm. The images are interpreted morphologically and are not used for phase identification in the absence of EDS/diffraction data.

Representative SEM images for selected specimens containing 70, 80, and 90 wt.% recycled material are shown in Figure 14. The images reveal pore/discontinuity morphology and the surrounding microstructural constituents at higher spatial contrast than the LM overview images. They are used as qualitative morphological support only; because EDS and diffraction-based phase identification were not performed for these observations, individual bright or elongated constituents are not assigned unambiguously to specific intermetallic phases.

In summary, architecture B achieved the lowest error for MeanPor. The *SF*_*Cr*0_-based representation remained competitive, achieved a consistently lower error than B for MostPor, and produced the nominally lowest RMSE for PoreArea. XAI analysis described the structure of the information used by the final models, whereas the V factor provided supplementary information on pore cross-sectional geometry.

## 4. Discussion

Recycled aluminum offers a clear energy advantage: the International Aluminium Institute reports that recycling may use about 95% less energy than primary production [5]. In our process, however, the higher recycled fraction was accompanied by a measurable quality penalty. Without intentional Mn correction, MeanPor rose from 0.160% at 50 wt.% recycled material to 6.111% at 95 wt.%. The recycled fraction therefore has to be considered together with metallurgical quality. These data do not define a universal “safe” recycling level, because the study did not include LCA, mechanical properties, or production rejection rates. Here, the models are used to distinguish process conditions by porosity risk. The observed trend agrees with earlier work on secondary Al-Si alloys; Závodská et al. [34], for example, reported increasing porosity with Fe content in AlSi7Mg0.3. Fe also rose almost in parallel with recycled material content in our dataset. Adding sample-specific Fe to Scrap + Mn treatment reduced RMSE from 0.3045 to 0.1691, making Fe a strong predictor of MeanPor. This predictive value should not be interpreted as proof that Fe is the sole cause of porosity. Recycled material content can also reflect changes in other alloying elements, melt history, oxides, hydrogen, and inclusions. Fe may therefore partly act as a marker of broader changes in charge quality. The earlier study of Piątkowski et al. [35] similarly showed that the balance between porosity mechanisms depends on the casting method and on filling and solidification conditions. Because hydrogen, bifilm, and oxide-inclusion content were not measured directly here, the observed porosity trend cannot be assigned to a single physical mechanism.

Mn correction targeted at Mn/Fe ≈ 0.5 lowered MeanPor over most of the studied range, but the size of the effect changed with recycled material content. The largest relative reduction occurred at 70 wt.%; the benefit was still present at 90–95 wt.% but was smaller. Previous work shows that Mn can suppress plate-like β-Al5FeSi-type phases and favor more compact α-Al(Fe,Mn)Si phases, with the outcome depending on Fe content, Mn/Fe ratio, and solidification conditions [20,21]. The LM images are consistent with a change in morphology after Mn correction, although elongated and plate-like precipitates remained visible at the highest recycled-material contents. Because no chemical microanalysis was performed, these precipitates were not assigned to specific phases. Thus, Mn/Fe ≈ 0.5 did not guarantee complete removal of unfavorable morphologies under the present conditions. The classical Sludge Factor, *SF* = (1 × %Fe) + (2 × %Mn) + (3 × %Cr), combines Fe, Mn, and Cr as an indicator of heavy-precipitate formation [32]. Sample-specific Cr was not measured, so the analysis used only *SF*_*Cr*0_= (1 × %Fe) + (2 × %Mn) and did not compare its absolute values with the literature thresholds for the full *SF*. Used alone, *SF*_*Cr*0_ predicted MeanPor poorly; combined with Scrap and Mn treatment, it reached R^2^ ≈ 0.986 and RMSE ≈ 0.208. This architecture gave the lowest RMSE for MostPor and the nominally lowest value for PoreArea. The MostPor advantage over B was stable in the bootstrap, whereas the PoreArea difference was not. These results support using *SF*_*Cr*0_ as a context-dependent Fe–Mn descriptor, not as a new physical “porosity factor.” For MeanPor, the best result came from second-order regression with predefined interactions. Kernel methods, GPR, tree ensembles, boosting, and MLP were also tested, but none improved generalization in this dataset. This finding is specific to the present data structure and should not be generalized to other casting problems. The 160 records represented only eight recycled-material levels, making LOSO important: each test fold contained a level absent from training rather than another replicate of a known level. One limitation is that outer CV also served to compare multiple candidates, so the reported performance of the selected model may include some selection optimism. Production use will require a frozen model and an independent campaign, and the equations should not be extrapolated beyond 60–95 wt.% or beyond the composition range represented here.

Accordingly, the reduction in porosity observed after Mn correction is not attributed to a single specific Fe-rich phase transformation. The LM and SEM observations are treated as qualitative morphological evidence. The SEM images provide additional higher-resolution visualization of pore/discontinuity morphology and surrounding constituents, but no EDS or diffraction-based phase identification was performed; elongated, plate-like, or bright constituents are therefore not assigned unambiguously to particular intermetallic phases. Hydrogen content, bifilm population, and oxide-inclusion quantity were not measured in this campaign, so the observed porosity trend cannot be assigned uniquely to one physical pore-formation mechanism. Direct SEM/EDS identification combined with quantitative measurements of intermetallic-particle morphology, size, and area fraction remains a priority for future mechanistic work.

LOSO and SHAP answered different questions in this work. LOSO assessed generalization, whereas SHAP described how the final models used the available predictors. In the present dataset, Scrap and Fe were strongly interdependent, while *SF*_*Cr*0_ was deterministically calculated from Fe and Mn. These are properties of the present data structure rather than claims derived from the external literature. More generally, Shapley-based attributions can depend strongly on how correlated or otherwise dependent predictors are handled [36,37]. Individual SHAP values therefore reflect model attribution, not the percentage contribution of a metallurgical mechanism. Grouped Shapley reduces some of the arbitrariness that arises with dependent features, but models B and E used different groupings, so their percentages were not compared directly. XAI was used to inspect model behavior and to generate hypotheses for further study [31,38]. The V analysis addressed a different aspect of porosity. Mean V increased from 0.68135 to 0.85935 after Mn correction at 90 wt.% recycled material and from 0.72135 to 0.91440 at 95 wt.%; MeanPor decreased in both cases. Because V was available for only part of the experimental plan, it was treated as supplementary. Its weak global correlation with MeanPor (r = 0.186) is consistent with pore amount and cross-sectional geometry describing different aspects of the defect. The V = 0.8 threshold remains auxiliary for this system, and neither V nor 2D LM images alone establish conversion of shrinkage porosity into gas porosity; dedicated discrimination methods using spatial and morphological information have been proposed for this purpose [39].

Model B also has a practical advantage: its main predictors—recycled material content, Mn-correction information, and Fe content—can potentially be obtained before casting. With independent validation, the model could support a preliminary porosity-risk screen; it is not a process-control model. The hierarchical structure of the dataset is an important limitation: the 160 chemistry–porosity records represent independently produced castings, but they are nested within 16 experimental conditions and only eight distinct recycled-material levels. The condition-level sensitivity analysis showed that the predictive metrics remained essentially unchanged after aggregation to 16 condition means, but this robustness check does not replace validation on independent production campaigns. Other limitations include the lack of sample-specific Cr, direct measurements of hydrogen, bifilms, and inclusion quantity, incomplete V coverage, strong dependence between recycled material content and chemical composition, and the single-site, single-process nature of the experimental campaign. Validation on independent production periods, equipment, or manufacturing sites is therefore required before broader industrial deployment. A further limitation concerns reproducibility of the previously completed computational analysis: the complete casting-level input matrix used for model fitting is not present in the currently retained documentation. The modeling metrics and previously reported summary statistics are preserved as outputs of those original calculations; they have not been independently recalculated from the specimen-level data during this revision.

## 5. Conclusions

The following conclusions were drawn from the results:

Across 50–95 wt.% recycled material, MeanPor increased nonlinearly. In the series without intentional Mn correction, the mean porosity area fraction rose from 0.160% to 6.111%.

Mn correction targeted at Mn/Fe ≈ 0.5 lowered MeanPor over most of the investigated range, but porosity still increased at the highest recycled-material contents. The largest relative reduction occurred at 70 wt.%; MeanPor at 95 wt.% remained 4.208%.

Among the evaluated sample-specific chemical descriptors, Fe added the most predictive information for MeanPor. Adding Fe to the Scrap + Mn treatment representation reduced RMSE from 0.3045 to 0.1691; model B reached R^2^ = 0.9910, RMSE = 0.1691, and MAE = 0.1508 in outer-grouped LOSO cross-validation.

A condition-level sensitivity analysis using only the 16 experimental condition means yielded R^2^ = 0.99100, RMSE = 0.16864, and MAE = 0.15088, essentially reproducing the primary 160-casting result. This supports the robustness of the within-campaign predictive trend while not constituting independent external validation.

*SF*_*Cr*0_ = (1 × %Fe) + (2 × %Mn) captured useful Fe–Mn information, although it is not the full Sludge Factor because sample-specific Cr was unavailable. For MeanPor, Scrap + Mn treatment + *SF*_*Cr*0_ achieved R^2^ ≈ 0.986 and RMSE ≈ 0.208. Fe gave the lower nominal error, but bootstrap analysis did not clearly separate the two representations.

Descriptor performance depended on the response variable. Fe was most informative for MeanPor, whereas the *SF*_*Cr*0_-based architecture gave the lowest RMSE for MostPor and PoreArea. The advantage over the Fe-based model was bootstrap-stable for MostPor but remained nominal for PoreArea.

Of the 13 model families tested, the explicit second-order regression with predefined interactions produced the lowest OOF error for MeanPor. Within the same architecture, its RMSE was consistently below that of Polynomial Ridge and MLP. Greater algorithmic complexity therefore did not reduce generalization error in this dataset.

SHAP indicated that the final models relied mainly on information associated with recycled material content and chemical composition. These attributions describe model behavior rather than the contribution of individual physical mechanisms, particularly for strongly interdependent features such as Scrap and Fe.

At 90 and 95 wt.% recycled material, Mn correction coincided with higher V and lower MeanPor. All available post-correction observations at these two levels had V ≥ 0.8. This threshold is only an auxiliary criterion for the present system, and the 2D results do not identify the pore-formation mechanism unambiguously.

Unlike single-correlation analysis or a simple fitted regression, the present workflow evaluates recycled material content, sample-specific chemistry, nonlinear terms, and interactions within one predictive framework. Outer-grouped LOSO cross-validation tests the models on entire recycled-material levels excluded from training, so the reported errors reflect prediction for unseen levels within the present experimental campaign rather than goodness of fit alone. This procedure should not be interpreted as independent external validation across different production campaigns, equipment, or manufacturing sites.

Following independent validation, the model could provide a faster and potentially less expensive preliminary assessment of porosity risk in LPC AlSi7Mg castings than full metallographic analysis, provided that comparable material and process conditions are maintained.

## Figures and Tables

**Table 1:** Summary of the experimental design and datasets used for modeling.

Item	Range/Variant
Alloy	AlSi7Mg
Technology	LPC
Scrap content	50–95 wt.% in 5 percentage-point increments
Mn—series 1	without intentional correction
Mn—series 2	correction to Mn/Fe ≈ 0.5
Replicates	10 independent wheel castings per condition
Full porosity dataset	200 observations (20 conditions × 10 independent castings)
Chemical composition + porosity dataset	160 observations (60–95 wt.%; 16 conditions × 10 independent castings)
Recycled feedstock source	Internal return scrap only: pre-production/test castings, rejected castings, machining chips, and gating-system returns; no externally purchased scrap

**Table 2:** Nominal chemical-composition specification of the base AlSi7Mg alloy.

Si	Fe	Cu	Mn	Mg	Zn	Ti	Ca	Sr	Other, Individual	Other, Total	Al
6.80–7.40	max. 0.13	max. 0.01	max. 0.03	0.26–0.32	max. 0.03	0.01–0.15	max. 0.002	0.02–0.03	0.03	0.01	bal.

**Table 3:** Parameters of liquid-metal preparation and the LPC process.

Parameter	Value/Status
Liquid-metal temperature before treatment	approximately 740 °C
Master alloy	AlTi5B1
Ti/B	5:1
Refining gas	N_2_
Refining time	approximately 10 min
Furnace model	Gas-fired shaft furnace for charge melting
Melt mass	20 metric tons
N_2_ flow rate/introduction method	18.5–19.5 L/min; bubbling
LPC machine	Buhler EcoLine S Pro (Bühler AG, Uzwil, Switzerland)
Die temperature	approximately 150 °C
Ladle preheat temperature	approximately 200 °C
Holding furnace	electric holding furnace; 690–700 °C
LPC pressure stages	approximately 200/500/800 mbar (20/50/80 kPa)
Casting/die cooling	casting: water; die: compressed cold air
Typical casting-cooling time	10–20 s, depending on casting size
Casting temperature after ejection	approximately 400 °C
Final water-cooling temperature	approximately 30–40 °C
Chemical-composition control	SpectroLab emission spectrometer
Density-index control	hydrostatic method; acceptable range 1–1.5%
Mn master alloy	AlMn20, 20 wt.% Mn, rod form; added ~15 min before casting
Mn target/auxiliary control	Mn/Fe ≈ 0.5; selected ATD checks of Tliq, TE, TEMn, Tsol
Separate filling/pressure-hold times	controlled by the fixed production recipe; separate numerical values not retained in the research documentation

**Table 4:** Metallographic preparation and microscopy procedure.

Item	Equipment/Procedure
Sectioning	Delta AbrasiMet Cutter 102511
Microscopy	LM–Olympus GX71MeF2
Images per sample	10 randomly selected fields of view
Etching	Porosity: unetched specimens; Fuss reagent only for selected qualitative LM observations
Identification of Fe-rich phases	Morphological assessment by LM; no direct chemical microanalysis
Additional SEM observations	Hitachi S-3400N; BSE mode; 200×; selected 70, 80, and 90 wt.% recycled-material specimens; morphology only, no EDS

**Table 5:** Variables describing porosity and the pore-morphology descriptor used in the study.

Variable	Unit	Interpretation/Status
MeanPor	%	mean pore area fraction from 10 fields of view; primary target variable
MostPor	%	maximum porosity area fraction among 10 fields of view
PoreArea	µm^2^	mean cross-sectional area of all pores identified in the 10 fields of a sample
V	–	dimensionless pore cross-sectional shape factor, V = 4πA/P^2^

**Table 6:** Predictor architectures evaluated in the chemical-composition-based modeling.

Code	Input Features	Purpose
A	Scrap + Mn treatment	process benchmark
B	Scrap + Mn treatment + Fe	incremental value of sample-specific Fe
C	Fe + Mn + Mg	chemistry-only architecture
D	Scrap + *SF*_*Cr*0_	scrap content + reduced Sludge Factor
E	Scrap + Mn treatment + *SF*_*Cr*0_	main architecture using *SF*
F	Scrap + Fe + Mn	direct Fe and Mn, without the binary Mn treatment variable
G	Scrap + Mn treatment + Fe + Mn	extended chemistry–process architecture
H	*SF*_*Cr*0_ only	standalone predictive value of *SF*

**Table 7:** Predictive model families and hyperparameter search ranges.

Model	Configuration Range
Second-order regression + predefined interactions	no tuning; terms defined a priori for the given architecture
PolynomialRidge	degree 2; StandardScaler; α = 0.001, 0.01, 0.1, 1, 10
PLS	n_components = 1–min(3,p)
GPR-RBF	Constant × ARD-RBF + WhiteKernel; normalize_y = True; 0 restarts
GPR-Matérn 5/2	Constant × ARD-Matérn(ν = 2.5) + WhiteKernel; 0 restarts
KernelRidge RBF	α = 0.03, 0.3, 3; γ = 0.03, 0.3, 3; scaling
SVR-RBF	C = 3, 30, 100; γ = 0.1, 1; ε = 0.05, 0.2; scaling
RandomForest	n_estimators = 120; max_depth = None/6; min_samples_leaf = 1/2; max_features = 1.0/sqrt
Extra Trees	n_estimators = 120; max_depth = None/6; min_samples_leaf = 1/2; max_features = 1.0/sqrt
Gradient Boosting	n_estimators = 120/200; learning_rate = 0.03/0.05; max_depth = 1/2
XGBoost	n_estimators = 150/250; learning_rate = 0.03/0.07; max_depth = 2; reg_lambda = 1/5; subsample = 0.85; colsample_bytree = 0.85
CatBoost	iterations = 200–300; depth = 3–4; learning_rate = 0.03/0.05; l2_leaf_reg = 3–10
MLP	L-BFGS; layers (8,), (16,), (16,8); α 0.001/0.01; max_iter = 2000

**Table 8:** Mean porosity area fraction as a function of recycled material content and Mn variant. Relative reduction was defined as 100 × (P_noMn − P_Mn)/P_noMn; a negative value indicates an increase in porosity after Mn correction.

Recycled Material [wt.%]	Without Mn Correction Mean [%]	After Mn Correction Mean [%]	Relative Reduction After Mn Correction [%]
50	0.160	0.180	−12.5
55	0.280	0.260	7.1
60	0.380	0.290	23.7
65	0.771	0.530	31.3
70	1.961	0.540	72.5
75	2.641	1.148	56.5
80	3.271	1.468	55.1
85	4.611	2.358	48.9
90	4.981	3.038	39.0
95	6.111	4.208	31.1

**Table 9:** Mean Fe, Mn, and *SF*_*Cr*0_ values for the common range of 60–95 wt.% recycled material.

Recycled Material [wt.%]	Fe Without Mn Correction [wt.%]	Mn Without Mn Correction [wt.%]	*SF*_*Cr*0_ Without Mn Correction	Fe After Mn Correction [wt.%]	Mn After Mn Correction [wt.%]	*SF*_*Cr*0_ After Mn Correction
60	0.1299	0.0199	0.1697	0.1399	0.0679	0.2757
65	0.1899	0.0299	0.2497	0.1899	0.1009	0.3917
70	0.2399	0.0199	0.2797	0.2399	0.1219	0.4837
75	0.3099	0.0299	0.3697	0.3199	0.1629	0.6457
80	0.3899	0.0299	0.4497	0.3999	0.2029	0.8057
85	0.4599	0.0299	0.5197	0.4599	0.2309	0.9217
90	0.6099	0.0199	0.6497	0.6199	0.3119	1.2437
95	0.6899	0.0099	0.7097	0.6999	0.3549	1.4097

**Table 10:** Explicit models A, B, and E used to assess the incremental value of chemical information.

Model	Features	R^2^	RMSE	MAE
A	Scrap + M	0.9707	0.3045	0.2591
B	Scrap + M + Fe	0.9910	0.1691	0.1508
E	Scrap + M + *SF*_*Cr*0_	0.9863	0.2081	0.1744

**Table 11:** Lowest OOF error obtained among the evaluated model families for each input architecture.

Rank	Arch.	Features	Best Model	R^2^	RMSE	MAE
1	B	Scrap + M + Fe	Quadratic + interactions	0.9910	0.1691	0.1508
2	E	Scrap + M + *SF*_*Cr*0_	PolynomialRidge	0.9863	0.2080	0.1771
3	F	Scrap + Fe + Mn	Quadratic + interactions	0.9857	0.2129	0.1974
4	G	Scrap + M + Fe + Mn	Quadratic + interactions	0.9853	0.2157	0.1826
5	D	Scrap + *SF*_*Cr*0_	Quadratic + interactions	0.9816	0.2412	0.2051
6	A	Scrap + M	KernelRidge RBF	0.9736	0.2890	0.2441
7	C	Fe + Mn + Mg	PolynomialRidge	0.9500	0.3978	0.3169
8	H	*SF*_*Cr*0_ only	PLS	0.0768	1.7087	1.4823

**Table 12:** Comparison of 13 model families for architectures B and E.

Model	B R^2^	B RMSE	B MAE	E R^2^	E RMSE	E MAE
Quadratic + interactions	0.9910	0.1691	0.1508	0.9863	0.2081	0.1744
PolynomialRidge	0.9838	0.2261	0.2022	0.9863	0.2080	0.1771
MLP Neural Network	0.9787	0.2597	0.2093	0.9633	0.3406	0.2852
KernelRidge RBF	0.9601	0.3552	0.2992	0.9776	0.2663	0.2287
GPR-Matérn 5/2	0.9309	0.4675	0.3827	0.8967	0.5716	0.4550
SVR-RBF	0.9282	0.4766	0.4044	0.9534	0.3840	0.3173
PLS	0.9187	0.5071	0.3829	0.8999	0.5627	0.4667
Extra Trees	0.9090	0.5366	0.4297	0.9097	0.5343	0.4190
CatBoost	0.8757	0.6269	0.4832	0.8341	0.7243	0.6035
XGBoost	0.8504	0.6879	0.5750	0.8296	0.7340	0.5757
RandomForest	0.8227	0.7489	0.5977	0.8330	0.7267	0.5436
Gradient Boosting	0.7834	0.8277	0.7065	0.7975	0.8003	0.6744
GPR-RBF	0.6374	1.0709	0.8581	0.6070	1.1149	0.9216

**Table 13:** Coefficients of the final explicit model B.

Term	Coefficient	Cluster-Robust SE	*p*
Intercept	−0.899543	1.469232	0.5495
Scrap	−0.155923	0.043187	0.00257
Mn treatment	12.939168	1.286508	4.63 × 10^−8^
Fe	−12.791573	2.018341	1.33 × 10^−5^
Scrap^2^	0.00340562	0.00041316	5.95 × 10^−7^
Scrap × M	−0.240276	0.023941	4.76 × 10^−8^
Fe × M	11.387443	1.523613	1.97 × 10^−6^

**Table 14:** Coefficients of the explicit model E.

Term	Coefficient	Cluster-Robust SE	*p*
Intercept	−5.176890	1.817294	0.0122
Scrap	−0.013324	0.050592	0.7959
Mn treatment	13.890001	1.672548	5.42 × 10^−7^
*SF*_*Cr*0_	−11.230108	2.060209	6.69 × 10^−5^
Scrap^2^	0.00229243	0.00043906	1.03 × 10^−4^
Scrap × M	−0.270059	0.032341	5.06 × 10^−7^
*SF*_*Cr*0_ × M	12.365551	1.760602	4.12 × 10^−6^

**Table 15:** OOF error of models B and E for individual held-out recycled-material levels.

Scrap [wt.%]	RMSE B	RMSE E	MAE B	MAE E
60	0.1051	0.1891	0.1008	0.1830
65	0.2002	0.2500	0.1965	0.2401
70	0.1812	0.2893	0.1776	0.2377
75	0.1240	0.0976	0.1109	0.0874
80	0.1975	0.1488	0.1920	0.1414
85	0.1060	0.3040	0.1021	0.2968
90	0.2202	0.1487	0.1665	0.1195
95	0.1753	0.1342	0.1600	0.1106

**Table 16:** Predictive value of individual additional chemical descriptors using an identical model structure.

Rank	X	R^2^	RMSE	MAE
1	Fe	0.9910	0.1691	0.1508
2	*SF*_*Cr*0_	0.9863	0.2081	0.1744
3	Si	0.9767	0.2714	0.2298
4	Cu	0.9734	0.2898	0.2424
5	Sr	0.9714	0.3008	0.2446
6	Mg	0.9695	0.3108	0.2664
7	Ca	0.9655	0.3301	0.2922
8	Mn	0.9646	0.3348	0.2839
9	Other	0.9645	0.3349	0.2619
10	Zn	0.9616	0.3486	0.3033
11	Ti	0.9439	0.4210	0.3613
12	Mn/Fe	0.9247	0.4881	0.3533

**Table 17:** Selected bootstrap comparisons of incremental feature value.

Comparison	Mean ΔRMSE	95% CI low	95% CI High	Replicates with Lower RMSE [%]
A quadratic → B Fe	0.1346	0.0706	0.2005	100.00
A quadratic → E *SF*	0.0977	0.0617	0.1379	100.00
E *SF* → B Fe	0.0371	−0.0294	0.1045	86.71
Si → *SF*_*Cr*0_	0.0647	0.0161	0.1136	99.52
Si → Fe	0.1015	0.0407	0.1563	99.94

**Table 18:** Comparison of explicit models with a common functional structure for three porosity descriptors.

Target	Arch.	Features	R^2^	RMSE	MAE	Rank
MeanPor	B	Scrap + M + Fe	0.9910	0.1691	0.1508	1
MeanPor	E	Scrap + M + *SF*_*Cr*0_	0.9863	0.2081	0.1744	2
MeanPor	F	Scrap + Fe + Mn	0.9857	0.2129	0.1974	3
MeanPor	G	Scrap + M + Fe + Mn	0.9853	0.2157	0.1826	4
MeanPor	D	Scrap + *SF*_*Cr*0_	0.9816	0.2412	0.2051	5
MeanPor	A	Scrap + M	0.9707	0.3045	0.2591	6
MeanPor	C	Fe + Mn + Mg	0.9386	0.4407	0.3614	7
MostPor	E	Scrap + M + *SF*_*Cr*0_	0.9511	0.7624	0.6404	1
MostPor	A	Scrap + M	0.9410	0.8373	0.6941	2
MostPor	G	Scrap + M + Fe + Mn	0.9410	0.8376	0.7028	3
MostPor	B	Scrap + M + Fe	0.9292	0.9171	0.7721	4
MostPor	D	Scrap + *SF*_*Cr*0_	0.9045	1.0650	0.9043	5
MostPor	F	Scrap + Fe + Mn	0.8830	1.1793	1.0003	6
MostPor	C	Fe + Mn + Mg	0.8528	1.3225	1.0228	7
PoreArea	E	Scrap + M + *SF*_*Cr*0_	0.9327	3.6033	2.6598	1
PoreArea	D	Scrap + *SF*_*Cr*0_	0.9145	4.0608	2.8208	2
PoreArea	B	Scrap + M + Fe	0.9122	4.1135	3.0679	3
PoreArea	A	Scrap + M	0.8791	4.8275	3.3185	4
PoreArea	G	Scrap + M + Fe + Mn	0.8791	4.8291	3.5389	5
PoreArea	F	Scrap + Fe + Mn	0.8756	4.8983	3.7150	6
PoreArea	C	Fe + Mn + Mg	0.8218	5.8610	5.0207	7

**Table 19:** Bootstrap comparisons of architecture E for the additional targets. The unit of ΔRMSE is p.p. for MostPor and µm^2^ for PoreArea.

Comparison	Mean ΔRMSE	95% CI Low	95% CI High	Replicates with Lower RMSE [%]
MostPor: A → E	0.0804	−0.1617	0.2909	75.17
MostPor: B → E	0.1536	0.0546	0.2538	99.97
PoreArea: A → E	1.1553	−0.2075	2.1133	95.87
PoreArea: B → E	0.5175	−0.0973	1.2525	92.77

**Table 20:** Global SHAP values for models B and E.

Model	Feature	Mean |SHAP| [p.p.]	Share [%]	95% CI Low	95% CI High
B quadratic	Scrap	2.5181	58.14	51.90	61.49
B quadratic	Fe	1.1383	26.28	22.69	28.21
B quadratic	Mn treatment	0.6745	15.57	10.56	25.26
E PolynomialRidge	Scrap	2.2817	64.87	58.36	68.38
E PolynomialRidge	*SF*_*Cr*0_	1.1429	32.49	28.92	39.20
E PolynomialRidge	Mn treatment	0.0930	2.64	2.39	2.82

**Table 21:** Grouped Shapley values.

Model	Block 1	Share 1 [%]	Block 2	Share 2 [%]
B quadratic	Scrap + Fe	67.24	Mn treatment	32.76
E PolynomialRidge	Scrap	67.74	*SF*_*Cr*0_ + Mn treatment	32.26

**Table 22:** Shape factor V under conditions with available sample-specific data.

Recycled Material [wt.%]	Series	*n*	Mean V	SD(V)	Mean Porosity [%]
70	without Mn correction	10	0.63240	0.00260	1.961
75	without Mn correction	10	0.58980	0.00520	2.641
80	without Mn correction	10	0.65995	0.00191	3.271
85	without Mn correction	10	0.63240	0.00260	4.611
90	without Mn correction	10	0.68135	0.00120	4.981
90	after Mn correction	10	0.85935	0.00120	3.038
95	without Mn correction	10	0.72135	0.00120	6.111
95	after Mn correction	10	0.91440	0.00260	4.208

**Table 23:** Comparison of V between Mn variants at 90 and 95 wt.% scrap.

Recycled Material [wt.%]	V Without Mn	V After Mn	ΔV	Welch *p*	Mann–Whitney *p*	Cohen d
90	0.68135	0.85935	+0.17800	1.63 × 10^−35^	1.79 × 10^−4^	148.0
95	0.72135	0.91440	+0.19305	6.44 × 10^−24^	1.80 × 10^−4^	95.3

## Data Availability

The data supporting the findings of this study are partially available from the corresponding author upon reasonable request. Access to certain data obtained in collaboration with an industrial partner is restricted for confidentiality reasons and may be provided only with permission from the relevant data owner.
